# Design, synthesis and antimalarial activity of novel bis{*N*-[(pyrrolo[1,2-*a*]quinoxalin-4-yl)benzyl]-3-aminopropyl}amine derivatives

**DOI:** 10.1080/14756366.2016.1268608

**Published:** 2017-01-23

**Authors:** Jean Guillon, Anita Cohen, Nassima Meriem Gueddouda, Rabindra Nath Das, Stéphane Moreau, Luisa Ronga, Solène Savrimoutou, Louise Basmaciyan, Alix Monnier, Myriam Monget, Sandra Rubio, Timothée Garnerin, Nadine Azas, Jean-Louis Mergny, Catherine Mullié, Pascal Sonnet

**Affiliations:** a ARNA Laboratory, University Bordeaux, UFR des Sciences Pharmaceutiques, Bordeaux, France;; b INSERM U1212, UMR CNRS 5320, ARNA Laboratory, Bordeaux, France;; c UMR-MD3, Faculty of Pharmacy, Aix-Marseille University, Laboratory of Parasitology, Marseille, France;; d Université de Picardie Jules Verne, Laboratoire de Glycochimie, des Antimicrobiens et des Agroressouces, UMR CNRS 7378, UFR de Pharmacie, Amiens, France

**Keywords:** Antimalarial activity, bis-pyrrolo[1,2-a]quinoxaline, *Plasmodium falciparum*, G-quadruplex, antileishmanial activity

## Abstract

Novel series of bis- and tris-pyrrolo[1,2-*a*]quinoxaline derivatives **1** were synthesized and tested for *in vitro* activity upon the intraerythrocytic stage of W2 and 3D7 *Plasmodium falciparum* strains. Biological results showed good antimalarial activity with IC_50_ in the μM range. In attempting to investigate the large broad-spectrum antiprotozoal activities of these new derivatives, their properties toward *Leishmania donovani* were also investigated and revealed their selective antiplasmodial profile. In parallel, the *in vitro* cytotoxicity of these molecules was assessed on the human HepG2 cell line. Structure–activity relationships of these new synthetic compounds are discussed here. The bis-pyrrolo[1,2-*a*]quinoxalines **1n** and **1p** were identified as the most potent antimalarial candidates with selectivity index (SI) of 40.6 on W2 strain, and 39.25 on 3D7 strain, respectively. As the telomeres of the parasite could constitute an attractive target, we investigated the possibility of targeting *Plasmodium* telomeres by stabilizing the *Plasmodium* telomeric G-quadruplexes through a FRET melting assay by our new compounds.

## Introduction

Malaria remains as one of the most devastating infectious diseases of the developing world. Malaria remains a major cause of public health problem in about 95 countries mainly located in the tropical zone of the globe (notably Africa, South-East Asia and also Eastern Mediterranean region)^1^; while approximately 3.2 billion people are at risk of being infected with malaria and developing disease. The latest figures on the incidence and mortality of malaria show that, despite progress in the implementation of preventive measures such as insecticide-treated mosquito nets and intermittent preventive treatments, this parasitic disease is still estimated to affect over 214 million people and to account for 438,000 deaths in 2015, of which approximately 80% are concentrated in just fifteen countries, mainly in Africa. The death toll is particularly high in children under five and pregnant women of the World Health Organization (WHO) African region^1^. Five species of protozoan parasites belonging to the *Plasmodium* genus, namely, *falciparum*, *malariae*, *vivax*, *ovale* and *knowlesi* cause malaria in human beings; from which, *P. falciparum* is the most dangerous of these species^2–4^.

The increasing prevalence of multiple drug resistant strains of *P. falciparum* in most malaria endemic areas has significantly reduced the efficacy of the current antimalarial drugs. Nowadays, the only fully effective antimalarial drugs utilize artemisinin and its derivatives, notably parenteral artesunate now recommended as the first line of treatment of severe malaria for at least 24 h and until oral medication could be tolerated^5^. Artemisinin and its derivatives are also used in combination with several different partner drugs (including lumefantrine, mefloquine, amodiaquine, sulfadoxine/pyrimethamine and piperaquine) in artemisinin-based combination therapies (ACTs), now recommended as the first line of treatment of uncomplicated *Plasmodium falciparum* malaria and as the second part of treatment for 3 days of severe malaria in endemic areas^5^. However, over the last decade evidence has grown that artemisinin resistance has emerged and spread within Southeast Asia, first on the Cambodia-Thailand border in 2009^6^^,^^7^, but now across a widening area of the Greater Mekong Subregion. Rapid scientific advances in understanding of this problem have taken place within the last five years^8–10^ and defined mutations in “K13” gene of *P. falciparum* associated to reduced ring-stage susceptibility to artemisinins^11^. Therefore, new antimalarial agents with new mechanisms of action are required to overcome the emergence of resistance and to control an ever-increasing number of epidemics caused by the malaria parasite^12^. Although current efforts in antimalarial drug discovery are focused on identification of new biological targets, continued research on new 4-aminoquinoline derivatives is still warranted. This is because the hemoglobin degradation pathway in *P. falciparum* has a proven history as an excellent therapeutic target to which the parasite has difficulty in evolving resistance. In spite of resistance to chloroquine, previous work has shown that modification of the lateral side chain of chloroquine results in aminoquinoline derivatives that avoid the chloroquine resistance mechanism^13^^,^^14^.

**Figure 1.**
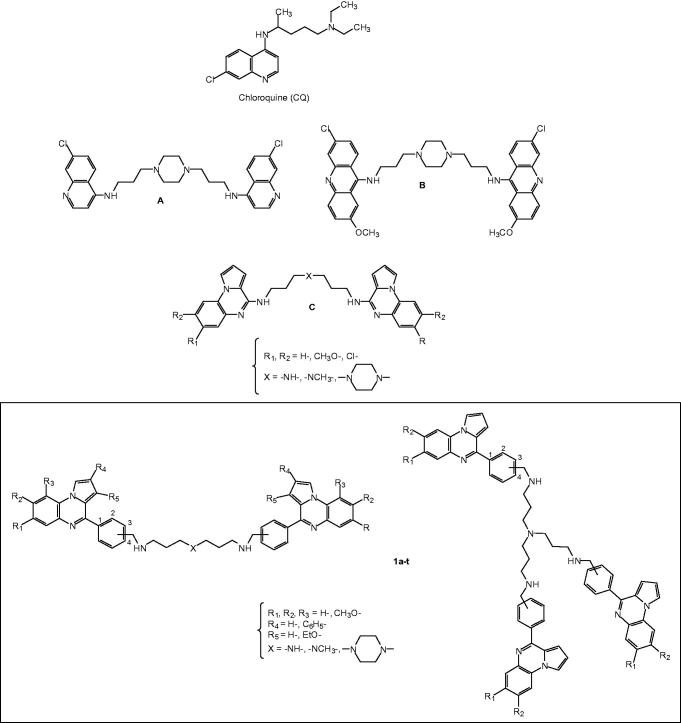
Structure of chloroquine, bisquinolines A, bisacridines B, bispyrrolo[1,2-*a*]quinoxalines C, and new synthesized substituted bis- and trispyrrolo[1,2-*a*]quinoxaline derivatives **1a–t**.

A possibility to overcome this multidrug-resistant mechanism is to design new quinoline-based drugs which will not be recognized by the protein system involved in drug efflux. In this regard, two series of compounds that show promise in this regard are the bisquinoline and bisacridine antimalarial drugs A and B (Figure 1)^15–18^. These drugs show much lower resistance indices than chloroquine, indicating that the bisquinoline or bisacridine structures are less efficiently excluded by drug-resistant parasites.

The pyrrolo[1,2-*a*]quinoxaline heterocyclic framework constitutes the basis of an important class of compounds possessing interesting biological activities. These compounds have been reported as key intermediates for the assembly of several heterocycles including antipsychotic agents, anti-HIV agents, adenosine A_3_ receptor modulators^19^, antiparasitic agents^20–25^ and antitumor agents^26–30^. In the course of our work devoted to discover new compounds employed in the antiparasitic chemotherapy, we previously identified some series of substituted pyrrolo[1,2-*a*]quinoxaline derivatives designed as interesting bioactive isosteres of quinoline derivatives^20–24^.Thus, taking into account our experience in the field of the synthesis of new antimalarial heterocyclic compounds based on our pyrrolo[1,2-*a*]quinoxaline heterocyclic core^20–24^^,^^27–30^, we decided to incorporate a benzyl group in position 4 of the heterocyclic skeletonof our previously described bispyrrolo[1,2-*a*]quinoxalines C^20^ to broaden the structural diversity of these derivatives, and mainly to increase the aromatic surface of these designed compounds (Figure 1).

Hence, we described here the synthesis of new bis- or trispyrrolo[1,2-*a*]quinoxaline derivatives **1a–t** (Figure 1) and reported on their *in vitro* antiplasmodial activity against the chloroquine-sensitive (3D7) and the chloroquine-resistant (W2) strains of the malaria parasite *Plasmodium falciparum*. As these new compounds were designed as quinoline-like bio-isosteres, and as the quinoline skeleton is the fundamental unit of many antiprotozoan drugs, these pyrrolo[1,2-*a*]quinoxaline derivatives were also investigated on another medically important protozoan, *Leishmania donovani*, in order to evaluate the specificity of their antiparasitic activity. Leishmaniasis is an infectious disease caused by protozoan parasites belonging to the genus *Leishmania*. *Leishmania* parasites exist in two major morphological stages: extracellular flagellated promastigotes in the digestive tract of their sandfly vector, which is the infective stage and immobile intracellular amastigotes in the cells of their host’ mononuclear phagocytic system. Leishmaniasis presents various clinical aspects including cutaneous leishmaniasis, the most common form, muco-cutaneous leishmaniasis and visceral leishmaniasis, the most severe form, lethal in untreated patients. *Leishmania donovani* is one of the major causative agents of human visceral leishmaniasis which represents a public health problem: 0.2 to 0.4 million visceral leishmaniasis cases occur each year and more than 90% of global visceral leishmaniasis cases occur in just five countries: India, Bangladesh, Sudan, Brazil and Ethiopa^31^. The current treatment of the disease is based on a limited number of chemotherapeutic agents (meglumine antimoniate, sodium stibogluconate, pentamidine, amphotericin B and miltefosine) which present many limits, notably characterized by a high toxicity and cost^32^^,^^33^. In addition, the *in vitro* cytotoxicity of these new molecules was assessed on the human HepG2 cell line, in order to determine their selectivity index. Moreover, as the telomeres of the parasite could constitute an attractive target, we also investigated the possibility of targeting *Plasmodium* telomeres by stabilizing the *Plasmodium* telomeric G-quadruplexes through a FRET melting assay by our new synthesized compounds. Indeed, telomerase activity has been identified in gametocytes and during the transition to erythrocytic stage of *P. falciparum*^34^. The telomeric 3' G-overhang region of *P. falciparum* is comprised of repeated degenerate unit ^5′^GGGTTYA^3′^ (where Y may be T or C)^35^ which can fold into intramolecular G-quadruplex^36^.

## Materials and methods

### Chemistry

Commercial reagents were used as received without additional purification. Melting points were determined with an SM-LUX-POL Leitz hot-stage microscope (Leitz GMBH, Midland, ON) and are uncorrected. IR spectra were recorded on a NICOLET 380FT-IR spectrophotometer (Thermo Electron Scientific Instruments LLC, Madison, WI). NMR spectra were recorded with tetramethylsilane as an internal standard using a BRUKER AVANCE 300 spectrometer (Bruker BioSpin, Wissembourg, France). Splitting patterns have been designated as follows: s = singlet; bs = broad singlet; d = doublet; t = triplet; q = quartet; qt = quintet, dd = double doublet; ddd = double double doublet; dt = double triplet; m = multiplet. Analytical TLC were carried out on 0.25 precoated silica gel plates (POLYGRAM SIL G/UV_254_) and visualization of compounds after UV light irradiation. Silica gel 60 (70–230 mesh) was used for column chromatography. Microwave experiments were carried out using a focused microwave reactor (CEM Discover, Saclay, France). High resolution mass spectra (electrospray in positive mode, ESI+) were recorded on a Waters Q-TOF Ultima apparatus. Mass spectra were recorded on an Ultraflex III TOF/TOF system (Bruker Daltonics, Bremen, Germany), equipped with 200 Hz smartbeam laser (355 nm) and operating in reflectron positive ion mode. Mass spectra were acquired over the m/z range 300–5000 by accumulating data from 1000 laser shots for each spectrum. The instrumental conditions employed to analyze molecular species were the following: ion source 1: 25.08 kV; ion source 2: 21.98 kV, lens: 11.03 kV, pulsed ion extraction: 30 ns, reflector: 26.39 kV, reflector 2: 13.79 kV. Matrix suppression was activated by deflection mode: suppression up to 450 Da. Mass calibration was performed for each sample with a peptide calibration mixture (8206195, Peptide Calibration Standard, Bruker Daltonics). The instrument was controlled using Bruker's flexControl 3.4 software and mass spectra were analyzed in Bruker's FlexAnalysis 3.4 software (Bruker Daltonics, Billerica, MA).

### General procedure for the synthesis of 4-(pyrrolo[1,2-*a*]quinoxalin-4-yl)benzaldehydes and 3-(pyrrolo[1,2-*a*]quinoxalin-4-yl)benzaldehydes8

To suspension of 4-chloropyrrolo[1,2-*a*]quinoxaline **7** (3.3 mmol), and Pd(PPh_3_)_4_ (0.164 mmol) in a mixture of toluene/EtOH (50/3 mL) under nitrogen were added K_2_CO_3_ (3.6 mmol) and phenylboronic acid (3.6 mmol). The reaction mixture was refluxed for 24 h, and the cooled suspension was extracted with CH_2_Cl_2_ (3 × 70 mL). The organic layer was washed with a saturated solution of NaCl (90 mL), and the combined organic extracts were dried over sodium sulfate, filtered, and evaporated under reduced pressure. The crude residue was triturated in ethanol. The resulting precipitate was filtered, washed with ethanol, and purified by column chromatography on silica gel using dichloromethane as eluent gave the pure product **8**.

#### 4–(9-Methoxypyrrolo[1,2-a]quinoxalin-4-yl)benzaldehyde (8d)

Yellow crystals, Yield: 85%, mp =162–164 °C; IR ν_max_ (KBr)/cm^−^^1^ 1705 (CHO); ^1^H NMR δ (300 MHz, CDCl_3_) 10.15 (s, 1H, CHO), 8.90 (dd, 1H, *J* = 2.70 and 1.20 Hz, H-1), 8.17 (d, 2H, *J*= 8.40 Hz, H-2′ and H-6′), 8.08 (d, 2H, *J* = 8.40 Hz, H-3′ and H-5′), 7.70 (dd, 1H, *J* = 8.10 and 1.20 Hz, H-6), 7.42 (t, 1H, *J*= 8.10 Hz, H-7), 7.13 (dd, 1H, *J* = 8.10 and 1.20 Hz, H-8), 7.00 (dd, 1H, *J* = 3.90 and 1.20 Hz, H-3), 6.90 (dd, 1H, *J* = 3.90 and 2.70 Hz, H-2), 4.13 (s, 3H, OCH_3_). MALDI-TOF MS m/z [M + H]^+ ^Calcd for C_19_H_15_N_2_O_2_: 303.113, Found: 303.127.

#### 4–(3-Ethoxypyrrolo[1,2-a]quinoxalin-4-yl)benzaldehyde (8e)

Yellow crystals, Yield: 68%, mp =170–172 °C; IR ν_max_ (KBr)/cm^−^^1^ 1700 (CHO); ^1^H NMR δ (300 MHz, CDCl_3_) 10.14 (s, 1H, CHO), 8.00–7.98 (m, 4H, H-2′, H-3′, H-5′ and H-6′), 7.94 (dd, 1H, *J* = 8.10 and 1.40 Hz, H-6), 7.84 (d, 1H, *J* = 3.15 Hz, H-1), 7.81 (dd, 1H, *J*= 8.10 and 1.40 Hz, H-9), 7.50 (ddd, 1H, *J* = 8.10, 8.10 and 1.40 Hz, H-7), 7.43 (ddd, 1H, *J* = 8.10, 8.10 and 1.40 Hz, H-8), 6.54(d, 1H, *J* = 3.15 Hz, H-2), 3.97(q, 2H, *J* = 6.90 Hz, OCH_2_), 1.17 (t, 3H, *J* = 6.90 Hz, CH_3_). MALDI-TOF MS m/z [M + H]^+ ^Calcd for C_20_H_17_N_2_O_2_: 317.129, Found: 317.135.

#### 3-(Pyrrolo[1,2-a]quinoxalin-4-yl)benzaldehyde (8 g)

Yellow crystals, Yield: 68%, mp =147–150 °C; IR ν_max_ (KBr)/cm^−^^1^ 1700 (CHO); ^1^H NMR δ (300 MHz, CDCl_3_) 10.18 (s, 1H, CHO), 8.56 (s, 1H, H-2′), 8.32 (d, 1H, *J* = 7.80 Hz, H-6), 8.09 (dd, 1H, *J* = 2.85 and 1.30 Hz, H-1), 8.08–8.05 (m, 2H, H-4′ and H-6′), 7.93 (d, 1H, *J* = 7.80 Hz, H-9), 7.75 (t, 1H, *J* = 7.65 Hz, H-5′), 7.56 (t, 1H, *J* = 7.80 Hz, H-7), 7.53 (t, 1H, *J* = 7.80 Hz, H-8), 7.02 (dd, 1H, *J* = 3.90 and 1.30 Hz, H-3), 6.96 (dd, 1H, *J* = 3.90 and 2.85 Hz, H-2). MALDI-TOF MS m/z [M + H]^+ ^Calcd for C_18_H_13_N_2_O: 273.103, Found: 273.127.

#### 3–(8-Methoxypyrrolo[1,2-a]quinoxalin-4-yl)benzaldehyde (8 h)

Beige crystals, Yield: 87%, mp =135–138 °C; IR ν_max_ (KBr)/cm^−^^1^ 1705 (CHO); ^1^H NMR δ (300 MHz, CDCl_3_) 10.16 (s, 1H, CHO), 8.54 (dd, 1H, *J* = 1.50 and 1.50 Hz,H-2′), 8.30 (ddd, 1H, *J* = 7.65, 1.50 and 1.50 Hz, H-4′), 8.05 (ddd, 1H, *J* = 7.65, 1.50 and 1.50 Hz, H-6′), 8.02 (d, 1H, *J* = 9.15 Hz, H-6), 7.95 (dd, 1H, *J* = 2.70 and 1.50 Hz, H-1), 7.73 (t, 1H, *J* = 7.65 Hz, H-5′),7.32 (d, 1H, *J* = 2.70 Hz, H-9), 7.10(dd, 1H, *J* = 9.15 and 2.70 Hz, H-7), 6.99 (dd, 1H, *J* = 3.90 and 1.50 Hz, H-3), 6.95 (dd, 1H, *J* = 3.90 and 2.70 Hz, H-2), 4.01 (s, 3H, CH_3_O). MALDI-TOF MS m/z [M + H]^+ ^Calcd for C_19_H_15_N_2_O_2_: 303.113, Found: 303.135.

### General procedure for bis{N-[(pyrrolo[1,2-*a*]quinoxalin-4-yl)benzylidene]-3-aminopropyl}methylamine and bis{N-[(pyrrolo[1,2-*a*]quinoxalin-4-yl)benzylidene]-3-aminopropyl}piperazine 9a-p, bis{N-[4-(indolo[1,2-*a*]quinoxalin-6-yl)benzylidene]-3-aminopropyl}methylamine 9q and bis{N-[(4-phenylpyrrolo[1,2-*a*]quinoxalin-2-yl)methylidene]-3-aminopropyl}piperazine 9r

To a solution of diamine (2.7 mmol) in ethanol (15 mL) was added (pyrrolo[1,2-*a*]quinoxalin-4-yl)benzaldehyde **8a–h** or (indolo[1,2-*a*]quinoxalin-6-yl)benzaldehyde **8i** or 4-phenylpyrrolo[1,2-*a*]quinoxaline-2-carboxaldehyde **8j** (5.4 mmol). The reaction mixture was then heated under reflux for 5 h and then evaporated to dryness under reduced pressure. After cooling, the residue was extracted with dichloromethane (40 mL). The organic layer was dried over sodium sulfate and evaporated to dryness. The products were then used without further purification.

#### Bis{N-[4-(pyrrolo[1,2-a]quinoxalin-4-yl)benzylidene]-3-aminopropyl}amine (9a)

Yellow crystals, Yield: 93%, mp =107–109 °C; ^1^H NMR δ (300 MHz, CDCl_3_) 8.31 (s, 2H, 2 HC = N), 8.03–7.80 (m, 8H, 2 H-2′, 2 H-6′, 2 H-6 and 2 H-1), 7.63 (d, 4H, *J* = 8.40 Hz, 2 H-3′ and 2 H-5′), 7.54–7.34 (m, 4H, 2 H-7 and 2 H-8), 6.96 (dd, 2H, *J* = 4.05 and 1.30 Hz, 2 H-3), 6.86 (dd, 2H, *J* = 4.05 and 2.70 Hz, 2 H-2), 4.09 (s, 1H, NH), 3.73–3.20 (m, 4H, 2 CH_2_), 2.88–2.31 (m, 4H, 2 CH_2_), 2.11–1.85 (m, 4H, 2 CH_2_).

#### Bis{N-[4-(pyrrolo[1,2-a]quinoxalin-4-yl)benzylidene]-3-aminopropyl}methylamine (9b)

Yellow oil, Yield: 89%; ^1^H NMR δ (300 MHz, CDCl_3_) 8.41 (s, 2H, 2 HC = N), 8.05 (d, 4H, *J* = 8.40 Hz, 2 H-2′ and 2 H-6′), 8.02 (dd, 2H, *J* = 8.25 and 1.40 Hz, 2 H-6), 7.97 (dd, 2H, *J* = 2.70 and 1.30 Hz, 2 H-1), 7.90 (d, 4H, *J* = 8.40 Hz, 2 H-3′ and 2 H-5′), 7.85 (dd, 2H, *J* = 8.25 and 1.40 Hz, 2 H-9), 7.52 (ddd, 2H, *J* = 8.25, 8.25 and 1.40 Hz, 2 H-7), 7.44 (ddd, 2H, *J* = 8.25, 8.25 and 1.40 Hz, 2 H-8),6.96 (dd, 2H, *J* = 4.05 and 1.30 Hz, 2 H-3), 6.87 (dd, 2H, *J* = 4.05 and 2.70 Hz, 2 H-2), 3.74 (t, 4H,*J* = 7.10 Hz, 2 CH_2_), 2.53 (t, 4H, *J* = 7.10 Hz, 2 CH_2_), 2.32 (s, 3H, CH_3_), 1.96 (qt, 4H, *J* = 7.10 Hz, 2 CH_2_).

#### Bis{N-[4-(pyrrolo[1,2-a]quinoxalin-4-yl)benzylidene]-3-aminopropyl}piperazine (9c)

Pale-yellow crystals, Yield: 78%, mp =177–180 °C; ^1^H NMR δ (300 MHz, CDCl_3_) 8.41 (s, 2H, 2 HC = N), 8.07 (d, 4H, *J* = 8.40 Hz, 2 H-2′ and 2 H-6′), 8.04 (dd, 2H, *J* = 8.10 and 1.20 Hz, 2 H-6), 7.97 (dd, 2H, *J* = 2.70 and 1.25 Hz, 2 H-1), 7.91 (d, 4H, *J* = 8.40 Hz, 2 H-3′ and 2 H-5′), 7.90 (dd, 2H, *J* = 8.10 and 1.20 Hz, 2 H-9), 7.55 (ddd, 2H, *J* = 8.10, 8.10 and 1.20 Hz, 2 H-7), 7.49 (ddd, 2H, *J* = 8.10, 8.10 and 1.20 Hz, 2 H-8), 7.01 (dd, 2H, *J* = 3.90 and 1.25 Hz, 2 H-3), 6.93 (dd, 2H, *J* = 3.90 and 2.70 Hz, 2 H-2), 3.73 (t, 4H, *J* = 6.90 Hz, 2 CH_2_), 2.78–2.50 (m, 8H, 4 CH_2_ pip.), 2.54 (t, 4H, *J* = 6.90 Hz, 2 CH_2_), 1.99 (qt, 4H, *J* = 6.90 Hz, 2 CH_2_).

#### Bis{N-[4–(7-methoxypyrrolo[1,2-a]quinoxalin-4-yl)benzylidene]-3-aminopropyl}methylamine (9d)

Yellow crystals, Yield: 96%, mp =67–69 °C; ^1^H NMR δ (300 MHz, CDCl_3_) 8.41 (s, 2H, 2 HC = N), 8.03 (d, 4H, *J* = 8.10 Hz, 2 H-2′ and 2 H-6′), 7.91 (dd, 2H, *J* = 2.70 and 1.20 Hz, 2 H-1), 7.89 (d, 4H, *J* = 8.10 Hz, 2 H-3′ and 2 H-5′), 7.78(d, 2H, *J* = 9.00 Hz, 2 H-9), 7.49 (d, 2H, *J* = 2.90 Hz, 2 H-6), 7.14 (dd, 2H, *J* = 9.00 and 2.90 Hz, 2 H-8), 6.94 (dd, 2H, *J* = 4.20 and 1.20 Hz, 2 H-3), 6.85 (dd, 2H, *J* = 4.20 and 2.70 Hz, 2 H-2), 3.93 (s, 6H, 2 CH_3_O), 3.74 (t, 4H, *J* = 6.90 Hz, 2 CH_2_), 2.52 (t, 4H, *J* = 6.90 Hz, 2 CH_2_), 2.31 (s, 3H, CH_3_), 1.96 (qt, 4H, *J* = 6.90 Hz, 2 CH_2_).

#### Bis{N-[4–(7-methoxypyrrolo[1,2-a]quinoxalin-4-yl)benzylidene]-3-aminopropyl}piperazine (9e)

Yellow crystals, Yield: 96%, mp =78–80 °C; ^1^H NMR δ (300 MHz, CDCl_3_) 8.41 (s, 2H, 2 HC = N), 8.07 (d, 4H, *J* = 8.20 Hz, 2 H-2′ and 2 H-6′), 7.97 (dd, 2H, *J* = 2.70 and 1.20 Hz, 2 H-1), 7.91 (d, 4H, *J* = 8.20 Hz, 2 H-3′ and 2 H-5′), 7.83 (d, 2H, *J* = 9.00 Hz, 2 H-9), 7.53 (d, 2H, *J* = 3.00 Hz, 2 H-6), 7.17 (dd, 2H, *J* = 9.00 and 3.00 Hz, 2 H-8), 6.99 (dd, 2H, *J* = 3.90 and 1.20 Hz, 2 H-3), 6.90 (dd, 2H, *J* = 3.90 and 2.70 Hz, 2 H-2), 3.95 (s, 6H, 2 CH_3_O), 3.73 (t, 4H, *J* = 6.90 Hz, 2 CH_2_), 2.71–2.45 (m, 8H, 4 CH_2_ pip.), 2.50 (t, 4H, *J* = 6.90 Hz, 2 CH_2_), 1.97 (qt, 4H, *J* = 6.90 Hz, 2 CH_2_).

#### Bis{N-[4–(8-methoxypyrrolo[1,2-a]quinoxalin-4-yl)benzylidene]-3-aminopropyl}methylamine (9f)

Yellow crystals, Yield: 96%, mp =61–63 °C; ^1^H NMR δ (300 MHz, CDCl_3_) 8.40 (s, 2H, 2 HC = N), 8.02 (d, 4H, *J* = 8.20 Hz, 2 H-2′ and 2 H-6′), 7.94 (d, 2H, *J* = 9.00 Hz, 2 H-6), 7.88(d, 4H, *J* = 8.20 Hz, 2 H-3′ and 2 H-5′), 7.87 (dd, 2H, *J* = 2.75 and 1.20 Hz, 2 H-1), 7.26 (d, 2H, *J* = 2.70 Hz, 2 H-9), 7.05 (dd, 2H, *J* = 9.00 and 2.70 Hz, 2 H-7), 6.95 (dd, 2H, *J* = 3.90 and 1.20 Hz, 2 H-3), 6.86 (dd, 2H, *J* = 3.90 and 2.75 Hz, 2 H-2), 3.99 (s, 6H, 2 CH_3_O), 3.73 (t, 4H, *J* = 6.90 Hz, 2 CH_2_), 2.51 (t, 4H, *J* = 6.90 Hz, 2 CH_2_), 2.31 (s, 3H, CH_3_), 1.96 (qt, 4H, *J* = 6.90 Hz, 2 CH_2_).

#### Bis{N-[4–(8-methoxypyrrolo[1,2-a]quinoxalin-4-yl)benzylidene]-3-aminopropyl}piperazine (9 g)

Pale-yellow crystals, Yield: 97%, mp =218–221 °C; ^1^H NMR δ (300 MHz, CDCl_3_) 8.40 (s, 2H, 2 HC = N), 8.06 (d, 4H, *J* = 8.40 Hz, 2 H-2′ and 2 H-6′), 7.99 (d, 2H, *J* = 9.00 Hz, 2 H-6), 7.93 (dd, 2H, *J* = 2.70 and 1.20 Hz, 2 H-1), 7.90 (d, 4H, *J*= 8.40 Hz, 2 H-3′ and 2 H-5′), 7.31 (d, 2H, *J* = 2.70 Hz, 2 H-9), 7.09 (dd, 2H, *J* = 9.00 and 2.70 Hz, 2 H-7), 6.98 (dd, 2H, *J* = 4.05 and 1.20 Hz, 2 H-3), 6.93 (dd, 2H, *J* = 4.05 and 2.70 Hz, 2 H-2), 4.00 (s, 6H, 2 CH_3_O), 3.73 (t, 4H, *J* = 6.90 Hz, 2 CH_2_), 2.72–2.43 (m, 8H, 4 CH_2_ pip.), 2.53 (t, 4H, *J* = 6.90 Hz, 2 CH_2_), 1.98 (qt, 4H, *J* = 6.90 Hz, 2 CH_2_).

#### Bis{N-[4–(9-methoxypyrrolo[1,2-a]quinoxalin-4-yl)benzylidene]-3-aminopropyl}methylamine (9 h)

Yellow crystals, Yield: 96%, mp =62–64 °C; ^1^H NMR δ (300 MHz, CDCl_3_) 8.82 (dd, 2H, *J* = 2.75 and 1.45 Hz, 2 H-1), 8.41 (s, 2H, 2 HC = N), 8.03 (d, 4H, *J* = 8.25 Hz, 2 H-2′ and 2 H-6′), 7.90 (d, 4H, *J* = 8.25 Hz, 2 H-3′ and 2 H-5′), 7.66 (dd, 2H, *J* = 8.15 and 1.20 Hz, 2 H-8), 7.36 (t, 2H, *J* = 8.15 Hz, 2 H-7), 7.05 (dd, 2H, *J* = 8.15 and 1.20 Hz, 2 H-6), 6.98 (dd, 2H, *J* = 4.10 and 1.45 Hz, 2 H-3), 6.83 (dd, 2H, *J* = 4.10 and 2.75 Hz, 2 H-2), 4.09 (s, 6H, 2 CH_3_O), 3.73 (t, 4H, *J* = 6.90 Hz, 2 CH_2_), 2.51 (t, 4H, *J* = 6.90 Hz, 2 CH_2_), 2.31 (s, 3H, CH_3_), 1.96 (qt, 4H, *J* = 6.90 Hz, 2 CH_2_).

#### Bis{N-[4–(9-methoxypyrrolo[1,2-a]quinoxalin-4-yl)benzylidene]-3-aminopropyl}piperazine (9i)

Pale-yellow crystals, Yield: 94%, mp =79–82 °C; ^1^H NMR δ (300 MHz, CDCl_3_) 8.87 (dd, 2H, *J* = 2.70 and 1.35 Hz, 2 H-1), 8.40 (s, 2H, 2 HC = N), 8.05 (d, 4H, *J* = 8.25 Hz, 2 H-2′ and 2 H-6′), 7.90 (d, 4H, *J* = 8.25 Hz, 2 H-3′ and 2 H-5′), 7.69 (dd, 2H, *J* = 8.10 and 1.20 Hz, 2 H-8), 7.40 (t, 2H, *J* = 8.10 Hz, 2 H-7), 7.09 (dd, 2H, *J* = 8.10 and 1.20 Hz, 2 H-6), 7.00 (dd, 2H, *J* = 4.20 and 1.35 Hz, 2 H-3), 6.87 (dd, 2H, *J* = 4.20 and 2.70 Hz, 2 H-2), 4.12 (s, 6H, 2 CH_3_O), 3.72 (t, 4H, *J* = 6.90 Hz, 2 CH_2_), 2.68–2.44 (m, 8H, 4 CH_2_ pip.), 2.52 (t, 4H, *J* = 6.90 Hz, 2 CH_2_), 1.98 (qt, 4H, *J* = 6.90 Hz, 2 CH_2_).

#### Bis{N-[4–(3-ethoxypyrrolo[1,2-a]quinoxalin-4-yl)benzylidene]-3-aminopropyl}methylamine (9j)

Yellow crystals, Yield: 97%, mp =37–39 °C; ^1^H NMR δ (300 MHz, CDCl_3_) 8.39 (s, 2H, 2 HC = N), 7.91 (dd, 2H, *J* = 7.80 and 1.50 Hz, 2 H-6), 7.86 (d, 4H, *J* = 8.40 Hz, 2 H-2′ and 2 H-6′), 7.82(d, 4H, *J* = 8.40 Hz, 2 H-3′ and 2 H-5′), 7.76 (d, 2H, *J* = 3.00 Hz, 2 H-1), 7.73 (dd, 2H, *J* = 7.80 and 1.50 Hz, 2 H-9), 7.43 (ddd, 2H, *J* = 7.80, 7.80 and 1.50 Hz, 2 H-7), 7.35 (ddd, 2H, *J* = 7.80, 7.80and 1.50 Hz, 2 H-8), 6.48 (d, 2H, *J* = 3.00 Hz, 2 H-2), 3.92 (q, 4H, *J* = 6.90 Hz, 2 OCH_2_), 3.72 (t, 4H, *J* = 6.90 Hz, 2 CH_2_), 2.50 (t, 4H, *J* = 6.90 Hz, 2 CH_2_), 2.29 (s, 3H, CH_3_), 1.95 (qt, 4H, *J* = 6.90 Hz, 2 CH_2_), 1.17 (t, 6H, *J* = 6.90 Hz, 2 CH_3_).

#### Bis{N-[4–(3-ethoxypyrrolo[1,2-a]quinoxalin-4-yl)benzylidene]-3-aminopropyl}piperazine (9k)

Yellow oil, Yield: 97%; ^1^H NMR δ (300 MHz, CDCl_3_) 8.38 (s, 2H, 2 HC = N), 7.93 (dd, 2H, *J*= 7.80 and 1.50 Hz, 2 H-6), 7.88 (d, 4H, *J* = 8.40 Hz, 2 H-2′ and 2 H-6′), 7.83 (d, 4H, *J* = 8.40 Hz, 2 H-3′ and 2 H-5′), 7.80 (d, 2H, *J* = 3.00 Hz, 2 H-1), 7.78 (dd, 2H, *J*= 7.80 and 1.50 Hz, 2 H-9), 7.47 (ddd, 2H, *J* = 7.80, 7.80 and 1.50 Hz, 2 H-7), 7.41 (ddd, 2H, *J* = 7.80, 7.80 and 1.50 Hz, 2 H-8), 6.53 (d, 2H, *J* = 3.00 Hz, 2 H-2), 3.96 (q, 4H, *J* = 6.90 Hz, 2 OCH_2_), 3.71 (t, 4H, *J* = 7.05 Hz, 2 CH_2_), 2.68–2.37 (m, 8H, 4 CH_2_ pip.), 2.48 (t, 4H, *J* = 7.05 Hz, 2 CH_2_), 1.96 (qt, 4H, *J* = 7.05 Hz, 2 CH_2_), 1.19 (t, 6H, *J* = 6.90 Hz, 2 CH_3_).

#### Bis{N-[4–(2-phenylpyrrolo[1,2-a]quinoxalin-4-yl)benzylidene]-3-aminopropyl}piperazine (9 l)

Orange crystals, Yield: 95%, mp =116–119 °C; ^1^H NMR δ (300 MHz, CDCl_3_) 8.43 (s, 2H, 2 HC = N), 8.29 (d, 2H, *J* = 1.50 Hz, 2 H-1), 8.11 (d, 4H, *J* = 8.40 Hz, 2 H-2′ and 2 H-6′), 8.07 (dd, 2H, *J* = 7.80 and 1.50 Hz, 2 H-6), 7.94 (dd, 2H, *J* = 7.80 and 1.50 Hz, 2 H-9),7.92 (d, 4H, *J* = 8.40 Hz, 2 H-3′ and 2 H-5′), 7.74–7.70 (m, 4H, 2 H-2” and 2 H-6”), 7.58 (ddd, 2H, *J* = 7.80, 7.80 and 1.50 Hz, 2 H-7), 7.51 (ddd, 2H, *J* = 7.80, 7.80 and 1.50 Hz, 2 H-8), 7.43 (t, 4H, *J* = 7.20 Hz, 2 H-3” and 2 H-5”), 7.36–7.30 (m, 2H, 2 H-4”), 7.25 (d, 2H, *J* = 1.50 Hz, 2 H-3), 3.74(t, 4H, *J* = 6.90 Hz, 2 CH_2_), 2.67–2.40 (m, 8H, 4 CH_2_ pip.), 2.52 (t, 4H, *J* = 6.90 Hz, 2 CH_2_), 1.99 (qt, 4H, *J* = 6.90 Hz, 2 CH_2_).

#### Bis{N-[3-(pyrrolo[1,2-a]quinoxalin-4-yl)benzylidene]-3-aminopropyl}methylamine (9m)

Yellow oil, Yield: 97%; ^1^H NMR δ (300 MHz, CDCl_3_) 8.43 (s, 2H, 2 HC = N), 8.30 (t, 2H, *J* = 1.50 Hz, 2 H-2′), 8.09–8.03 (m, 4H, 2 H-6 and 2 H-4′), 8.02 (dd, 2H, *J* = 2.70 and 1.35 Hz, 2 H-1), 7.93–7.86 (m, 4H, 2 H-6′ and 2 H-9), 7.63–7.44 (m, 4H, 2 H-5′, 2 H-7 and 2 H-8),7.03 (dd, 2H, *J* = 3.90 and 1.35 Hz, 2 H-3), 6.90 (dd, 2H, *J* = 3.90 and 2.70 Hz, 2 H-2), 3.71 (t, 4H, *J* = 6.90 Hz, 2 CH_2_), 2.47 (t, 4H, *J* = 6.90 Hz, 2 CH_2_), 2.29 (s, 3H, CH_3_), 1.93 (qt, 4H, *J* = 6.90 Hz, 2 CH_2_).

#### Bis{N-[3-(pyrrolo[1,2-a]quinoxalin-4-yl)benzylidene]-3-aminopropyl}piperazine (9n)

Yellow oil, Yield: 98%; ^1^H NMR δ (300 MHz, CDCl_3_) 8.42 (s, 2H, 2 HC = N), 8.32 (s, 2H, 2 H-2′), 8.09–8.03 (m, 6H, 2 H-6, 2 H-4′ and 2 H-1), 7.98–7.90 (m, 4H, 2 H-6′ and 2 H-9), 7.63–7.46 (m, 4H, 2 H-5′, 2 H-7 and 2 H-8), 7.03–7.01 (m, 2H, 2 H-3), 6.93–6.91 (m, 2H, 2 H-2), 3.71 (t, 4H, *J* = 6.90 Hz, 2 CH_2_), 2.65–2.42 (m, 8H, 4 CH_2_ pip.), 2.48 (t, 4H, *J* = 6.90 Hz, 2 CH_2_), 1.95 (qt, 4H, *J* = 6.90 Hz, 2 CH_2_).

#### Bis{N-[3–(8-methoxypyrrolo[1,2-a]quinoxalin-4-yl)benzylidene]-3-aminopropyl}methylamine (9o)

Yellow oil, Yield: 98%; ^1^H NMR δ (300 MHz, CDCl_3_) 8.41 (s, 2H, 2 HC = N), 8.29 (dd, 2H, *J* = 1.50 and 1.50 Hz, 2 H-2′), 8.03 (ddd, 2H, *J* = 7.35, 1.50 and 1.50 Hz, 2 H-4′), 7.97 (d, 2H, *J* = 9.00 Hz, 2 H-6), 7.93 (ddd, 2H, *J* = 7.35, 1.50 and 1.50 Hz, 2 H-6′), 7.89 (dd, 2H, *J* = 2.85 and 1.35 Hz, 2 H-1), 7.56 (t, 2H, *J* = 7.65 Hz, 2 H-5′), 7.30 (d, 2H, *J* = 2.70 Hz, 2 H-9), 7.07 (dd, 2H, *J* = 9.00 and 2.70 Hz, 2 H-7), 6.97 (dd, 2H, *J* = 3.90 and 1.35 Hz, 2 H-3), 6.89 (dd, 2H, *J* = 3.90 and 2.85 Hz, 2 H-2), 3.99 (s, 6H, 2 CH_3_O),3.71 (t, 4H, *J* = 7.05 Hz, 2 NCH_2_), 2.50 (t, 4H, *J* = 7.05 Hz, 2 NCH_2_), 2.30 (s, 3H, NCH_3_), 1.93 (qt, 4H, *J* = 7.05 Hz, 2 CH_2_).

#### Bis{N-[3–(8-methoxypyrrolo[1,2-a]quinoxalin-4-yl)benzylidene]-3-aminopropyl}piperazine (9p)

Orange oil, Yield: 55%; ^1^H NMR δ (300 MHz, CDCl_3_) 8.41 (s, 2H, 2 HC = N), 8.30 (dd, 2H, *J* = 1.50 and 1.50 Hz, 2 H-2′), 8.05 (ddd, 2H, *J* = 7.40, 1.50 and 1.50 Hz, 2 H-4′), 8.01 (d, 2H, *J* = 9.00 Hz, 2 H-6), 7.94 (ddd, 2H, *J* = 7.40, 1.50 and 1.50 Hz, 2 H-6′), 7.91 (dd, 2H, *J* = 2.70 and 1.35 Hz, 2 H-1), 7.59 (t, 2H, *J* = 7.40 Hz, 2 H-5′), 7.29 (d, 2H, *J* = 2.70 Hz, 2 H-9), 7.08 (dd, 2H, *J* = 9.00 and 2.70 Hz, 2 H-7), 6.98 (dd, 2H, *J* = 3.90 and 1.35 Hz, 2 H-3), 6.92 (dd, 2H, *J* = 3.90 and 2.70 Hz, 2 H-2), 4.00 (s, 6H, 2 CH_3_O),3.70 (t, 4H, *J* = 6.90 Hz, 2 NCH_2_), 2.62–2.42 (m, 8H, 4 NCH_2_ pip.), 2.48 (t, 4H, *J* = 6.90 Hz, 2 NCH_2_), 1.95 (qt, 4H, *J* = 6.90 Hz, 2 CH_2_).

#### Bis{N-[4-(indolo[1,2-a]quinoxalin-6-yl)benzylidene]-3-aminopropyl}methylamine (9q)

Yellow crystals, Yield: 84%, mp =226–229 °C; ^1^H NMR δ (300 MHz, CDCl_3_) 8.57 (d, 2H, *J* = 8.00 Hz, 2 H-11), 8.53 (d, 2H, *J* = 8.10 Hz, 2 H-1), 8.44 (s, 2H, 2 HC = N), 8.15–8.08 (m, 6H, 2 H-2′, 2 H-6′ and 2 H-4), 7.97–7.92 (m, 6H, 2 H-3′, 2 H-5′ and 2 H-8), 7.70–7.57 (m, 4H, 2 H-2 and 2 H-3), 7.51–7.45 (m, 4H, 2 H-9 and 2 H-10), 7.26 (s, 2H, 2 H-7), 3.76 (t, 4H, *J* = 6.90 Hz, 2 NCH_2_), 2.87–2.55 (m, 12H, 4 NCH_2_ pip. and 2 NCH_2_), 2.09 (qt, 4H, *J* = 6.90 Hz, 2 CH_2_).

#### Bis{N-[(4-phenylpyrrolo[1,2-a]quinoxalin-2-yl)methylidene]-3-aminopropyl}piperazine (9r)

Beige crystals, Yield: 60%, mp >250 °C; ^1^H NMR δ (300 MHz, CDCl_3_) 8.44 (s, 2H, 2 HC = N), 8.32 (d, 2H, *J* = 1.10 Hz, 2 H-1), 8.06 (dd, 2H, *J* =7.80 and 1.60 Hz, 2 H-6), 8.02–7.99 (m, 4H, 2 H-2′ and 2 H-6′), 7.90 (dd, 2H, *J* = 7.80 and 1.60 Hz, 2 H-9), 7.59–7.53 (m, 8H, 2 H-3′, 2 H-4′, 2 H-5′ and 2 H-7), 7.50 (ddd, 2H, *J* = 7.80, 7.80, 1.60 Hz, 2 H-8), 7.29 (d, 2H, *J* = 1.10 Hz, 2 H-3), 3.67 (t, 4H, *J* = 6.90 Hz, 2 NCH_2_), 2.69–2.43 (m, 8H, 4 NCH_2_ pip.), 2.47 (t, 4H, *J* = 6.90 Hz, 2 NCH_2_), 1.94 (qt, 4H, *J* = 6.90 Hz, 2 CH_2_).

### General procedure for tris{*N*-[(pyrrolo[1,2-*a*]quinoxalin-4-yl)benzylidene]-3-aminopropyl}amines 9s-t

To a solution of triamine (1.8 mmol) in ethanol (15 mL) was added (pyrrolo[1,2-*a*]quinoxalin-4-yl)benzaldehyde **8i–j** (5.4 mmol). The reaction mixture was then heated under reflux for 5 h and then evaporated to dryness under reduced pressure. After cooling, the residue was extracted with dichloromethane (40 mL). The organic layer was dried over sodium sulfate and evaporated to dryness. Products were then used without further purification.

#### Tris{N-[4–(7-methoxypyrrolo[1,2-a]quinoxalin-4-yl)benzylidene]-3-aminopropyl}amine (9s)

Orange oil, Yield: 82%; ^1^H NMR δ (300 MHz, CDCl_3_) 8.42 (s, 2H, 3 HC = N), 7.99 (d, 6H, *J* = 8.40 Hz, 3 H-2′ and 3 H-6′), 7.88 (d, 6H, *J* = 8.40 Hz, 3 H-3′ and 3 H-5′), 7.86 (dd, 3H, *J* = 2.70 and 1.20 Hz, 3 H-1), 7.73 (d, 3H, *J* = 9.00 Hz, 3 H-9), 7.45 (d, 3H, *J* = 2.80 Hz, 3 H-6), 7.11 (dd, 3H, *J* = 9.00 and 2.80 Hz, 3 H-8), 6.90 (dd, 3H, *J* = 4.05 and 1.20 Hz, 3 H-3), 6.80 (dd, 3H, *J* = 4.05 and 2.70 Hz, 3 H-2), 3.91 (s, 9H, 3 CH_3_O), 3.76 (t, 6H, *J* = 6.90 Hz, 3 CH_2_), 2.64 (t, 6H, *J* = 6.90 Hz, 3NCH_2_), 1.96 (qt, 6H, *J* = 6.90 Hz, 3 CH_2_).

#### Tris{N-[3–(8-methoxypyrrolo[1,2-a]quinoxalin-4-yl)benzylidene]-3-aminopropyl}amine (9t)

Orange oil, Yield: 71%; ^1^H NMR δ (300 MHz, CDCl_3_) 8.40 (s, 3H, 3 HC = N), 8.27 (dd, 3H, *J* = 1.50 and 1.50 Hz, 3 H-2′), 7.99 (ddd, 3H, *J* = 7.80, 1.50 and 1.50 Hz, 3 H-4′), 7.98(d, 3H, *J* = 9.00 Hz, 3H-6), 7.92 (ddd, 3H, *J* = 7.80, 1.50 and 1.50 Hz, 3H-6′), 7.86 (dd, 3H, *J* = 2.85 and 1.35 Hz, 3 H-1), 7.52 (t, 3H, *J* = 7.80 Hz, 3 H-5′), 7.25 (d, 3H, *J* = 2.70 Hz, 3 H-9), 7.04 (dd, 3H, *J* = 9.00 and 2.70 Hz, 3 H-7), 6.94 (dd, 3H, *J* = 4.05 and 1.35 Hz, 3 H-3), 6.87 (dd, 3H, *J* = 4.05 and 2.85 Hz, 3 H-2), 3.98 (s, 9H, 3 CH_3_O),3.71 (t, 6H, *J* = 6.90 Hz, 3 NCH_2_), 2.61 (t, 6H, *J* = 6.90 Hz, 3 NCH_2_), 1.92 (qt, 6H, *J* = 6.90 Hz, 3 CH_2_).

### N, N′- [oxybis(2, 1-ethanediyloxy-2, 1-ethanediyl)] bis-3–(8-methoxypyrrolo[1,2-*a*]quinoxalin-4-yl)benzylimine 9u

To a solution of 1,11-diamino-3,6,9-trioxaundecane (0.56 mmol) in ethanol (12 mL) was added 3-(pyrrolo[1,2-*a*]quinoxalin-4-yl)benzaldehyde **8j** (1.12 mmol). The reaction mixture was then heated under reflux for 5 h and then evaporated to dryness under reduced pressure. After cooling, the residue was extracted with dichloromethane (35 mL). The organic layer was dried over sodium sulfate and evaporated to dryness. Product **9u** was then used without further purification. Yellow oil, Yield: 97%; ^1^H NMR δ (300 MHz, CDCl_3_) 8.39 (s, 2H, 2 HC = N), 8.29 (dd, 2H, *J* = 1.50 and 1.50 Hz, 2 H-2′), 8.05–7.87 (m, H, 2 H-4′, 2 H-6, 2 H-6′ and 2 H-1), 7.59–7.55 (m, 2H, 2 H-5′),7.27 (d, 2H, *J* = 2.70 Hz, 2 H-9), 7.06 (dd, 2H, *J* = 9.00 and 2.70 Hz, 2 H-7), 6.96 (m, 2H, 2 H-3), 6.88 (m, 2H, 2 H-2), 3.95 (s, 6H, 2 CH_3_O), 3.81–3.79 (m, 8H, 4OCH_2_), 3.64–3.62 (m, 8H, 4 OCH_2_).

### General procedure for bis{N-[(pyrrolo[1,2-*a*]quinoxalin-4-yl)benzyl]-3-aminopropyl}methylamine and bis{N-[(pyrrolo[1,2-*a*]quinoxalin-4-yl)benzyl]-3-aminopropyl}piperazine 1a-p, bis{N-[4-(indolo[1,2-*a*]quinoxalin-6-yl)benzyl]-3-aminopropyl} piperazine 1q and bis{N-[(4-phenylpyrrolo[1,2-*a*]quinoxalin-2-yl)methyl]-3-aminopropyl}piperazine 1r and tris{N-[(pyrrolo[1,2-*a*]quinoxalin-4-yl)benzyl]-3-aminopropyl}amine 1s-t and N,N′-[oxybis(2,1-ethanediyloxy-2,1-ethanediyl)]bis-3–(8-methoxypyrrolo[1,2-*a*]quinoxalin-4-yl)benzylamine 1u

To a solution of compound **9** (1.26 mmol) in methanol (40 mL) was added portion-wise at 0 °C sodium borohydride (10.1 mmol; 8 equiv.). The reaction mixture was then heated under reflux for 4 h and then evaporated to dryness under reduced pressure. After cooling, the residue was triturated in water and extracted with dichloromethane (85 mL). The organic layer was separated, dried over sodium sulfate and evaporated to dryness. The residue were then purified by column chromatography on silica gel using dichloromethane/methanol (90/10, v/v) as eluent to give the pure product **1**.

#### Bis{N-[4-(pyrrolo[1,2-a]quinoxalin-4-yl)benzyl]-3-aminopropyl}amine (1a)

Yellow oil, Yield: 91%; ^1^H NMR δ (300 MHz, CDCl_3_) 8.02 (dd, 2H, *J* = 7.80 and 1.50 Hz, 2 H-6), 7.96 (d, 4H, *J* = 8.20 Hz, 2 H-2′ and 2 H-6′), 7.93 (dd, 2H, *J* = 2.70 and 1.20 Hz, 2 H-1), 7.82 (dd, 2H, *J* = 7.80 and 1.50 Hz, 2 H-9), 7.48 (d, 4H, *J* = 8.20 Hz, 2 H-3′ and 2 H-5′), 7.47–7.39 (m, 4H, 2 H-7 and 2 H-8), 6.98 (dd, 2H, *J* = 3.90 and 1.20 Hz, 2 H-3), 6.86 (dd, 2H, *J* = 3.90 and 2.70 Hz, 2 H-2), 3.89 (s, 4H, 2 NCH_2_), 3.73 (t, 4H, *J* = 6.90 Hz, 2 NCH_2_), 2.71(t, 4H, *J* = 6.90 Hz, 2 NCH_2_), 1.84 (bs, 3H, 3 NH), 1.77 (qt, 4H, *J* = 6.90 Hz, 2 CH_2_) ^13^C NMR δ (100 MHz, CDCl_3_) 155.3 (C-4), 141.8 (C-3a), 139.0 (C-5a), 138.7 (C-4′), 137.6 (C-1′), 131.5 (C-8), 130.3 (C-2′ and C-6′), 130.1 (C-3′ and C-5′), 128.8 (C-7), 128.5 (C-9a), 126.6 (C-6), 116.0 (C-1), 115.4 (C-2), 115.0 (C-9), 110.0 (C-3), 54.5 (NCH_2_), 50.0 (NCH_2_), 48.9 (NCH_2_), 27.7 (CH_2_). MALDI-TOF MS m/z [M + H]^+ ^Calcd for C_42_H_42_N_7_: 644.350, Found: 644.342.

#### Bis{N-[4-(pyrrolo[1,2-a]quinoxalin-4-yl)benzyl]-3-aminopropyl}methylamine (1b)

Orange oil, Yield: 94%; ^1^H NMR δ (300 MHz, CDCl_3_) 8.00 (dd, 2H, *J* = 8.00 and 1.35 Hz, 2 H-6), 7.96 (d, 4H, *J* = 8.40 Hz, 2 H-2′ and 2 H-6′), 7.95 (dd, 2H, *J* = 2.70 and 1.30 Hz, 2 H-1), 7.83 (dd, 2H, *J* = 8.00 and 1.35 Hz, 2 H-9), 7.49 (d, 4H, *J* = 8.40 Hz, 2 H-3′ and 2 H-5′), 7.47–7.39 (m, 4H, 2 H-7 and 2 H-8), 6.97 (dd, 2H, *J* = 3.90 and 1.30 Hz, 2 H-3), 6.86 (dd, 2H, *J* = 3.90 and 2.70 Hz, 2 H-2), 3.88 (s, 4H, 2 NCH_2_), 2.71 (t, 4H, *J* = 7.10 Hz, 2 NCH_2_), 2.43 (t, 4H, *J* = 7.10 Hz, 2 NCH_2_), 2.24 (s, 3H, NCH_3_), 1.73 (qt, 4H, *J* = 7.10 Hz, 2 CH_2_). ^13^C NMR δ (100 MHz, CDCl_3_) 155.2 (C-4), 140.2 (C-3a), 139.3 (C-5a), 139.2 (C-4′), 137.5 (C-1′), 131.5 (C-8), 130.4 (C-2′ and C-6′), 130.3 (C-3′ and C-5′), 128.8 (C-7), 128.4 (C-9a), 126.6 (C-6), 116.0 (C-1), 115.4 (C-2), 115.0 (C-9), 110.0 (C-3), 57.3 (NCH_2_), 54.1 (NCH_2_), 48.6 (NCH_2_), 43.4 (NCH_3_), 27.0 (CH_2_). MALDI-TOF MS m/z [M + H]^+ ^Calcd for C_43_H_44_N_7_: 658.366, Found: 658.362.

#### Bis{N-[4-(pyrrolo[1,2-a]quinoxalin-4-yl)benzyl]-3-aminopropyl}piperazine (1c)

Colorless oil, Yield: 61%; ^1^H NMR δ (300 MHz, CDCl_3_) 8.04 (dd, 2H, *J* = 8.25 and 1.35 Hz, 2 H-6), 8.00 (dd, 2H, *J* = 2.70 and 1.20 Hz, 2 H-1), 7.98 (d, 4H, *J* = 8.40 Hz, 2 H-2′ and 2 H-6′), 7.89 (dd, 2H, *J* = 8.25 and 1.35 Hz, 2 H-9), 7.53 (ddd, 2H, *J* = 8.25, 8.25 and 1.35 Hz, 2 H-7), 7.50 (d, 4H, *J* = 8.40 Hz, 2 H-3′ and 2 H-5′), 7.45 (ddd, 2H, *J* = 8.25, 8.25 and 1.35 Hz, 2 H-8),7.01 (dd, 2H, *J* = 3.90 and 1.20 Hz, 2 H-3), 6.91 (dd, 2H, *J* = 3.90 and 2.70 Hz, 2 H-2), 3.91 (s, 4H, 2 NCH_2_), 2.74 (t, 4H, *J* = 6.90 Hz, 2 NCH_2_), 2.58–2.39 (m, 8H, 4 NCH_2_ pip.), 2.44 (t, 4H, *J* = 6.90 Hz, 2 NCH_2_), 1.76 (qt, 4H, *J* = 6.90 Hz, 2 CH_2_). ^13^C NMR δ (100 MHz, CDCl_3_) 155.2 (C-4), 140.8 (C-3a), 139.3 (C-5a), 139.2 (C-4′), 137.6 (C-1′), 131.6 (C-8), 130.3 (C-2′ and C-6′), 130.2 (C-3′ and C-5′), 128.9 (C-7), 128.5 (C-9a), 126.7 (C-6), 116.1 (C-1), 115.4 (C-2), 115.0 (C-9), 110.0 (C-3), 58.5 (NCH_2_), 54.4 (NCH_2_), 54.2 (NCH_2_), 49.5 (NCH_2_), 26.5 (CH_2_). MALDI-TOF MS m/z [M + H]^+ ^Calcd for C_46_H_49_N_8_: 713.408, Found: 713.412.

#### Bis{N-[4–(7-methoxypyrrolo[1,2-a]quinoxalin-4-yl)benzyl]-3-aminopropyl}methylamine (1d)

Pale-yellow crystals, Yield: 88%, mp =65–67 °C; ^1^H NMR δ (300 MHz, CDCl_3_) 7.95 (d, 4H, *J* = 8.10 Hz, 2 H-2′ and 2 H-6′), 7.90 (dd, 2H, *J* = 2.70 and 1.20 Hz, 2 H-1), 7.76 (d, 2H, *J* = 9.00 Hz, 2 H-9), 7.49 (d, 2H, *J* = 2.70 Hz, 2 H-6), 7.48 (d, 4H, *J* = 8.10 Hz, 2 H-3′ and 2 H-5′), 7.11 (dd, 2H, *J* = 9.00 and 2.70 Hz, 2 H-8), 6.96 (dd, 2H, *J* = 4.05 and 1.20 Hz, 2 H-3), 6.84 (dd, 2H, *J* = 4.05 and 2.70 Hz, 2 H-2), 3.91 (s, 6H, 2 CH_3_O), 3.89 (s, 4H, 2 NCH_2_), 2.72 (t, 4H, *J* = 7.05 Hz, 2 NCH_2_), 2.43 (t, 4H, *J* = 7.05 Hz, 2 NCH_2_), 1.73 (qt, 4H, *J* = 7.05 Hz, 2 CH_2_). ^13^C NMR δ (100 MHz, CDCl_3_) 158.5 (C-7), 155.9 (C-4), 143.7 (C-5a), 138.7 (C-3a), 138.5 (C-4′), 130.1 (C-2′ and C-6′), 129.6 (C-3′ and C-5′), 126.4 (C-1′), 122.8 (C-9a), 117.9 (C-9), 115.9 (C-8), 115.6 (C-1), 115.0 (C-2), 112.6 (C-3), 109.7 (C-6), 57.6 (NCH_2_), 57.1 (OCH_3_), 55.2 (NCH_2_), 49.5 (NCH_2_), 43.7 (NCH_3_), 29.0 (CH_2_). MALDI-TOF MS m/z [M + H]^+ ^Calcd for C_45_H_48_N_7_O_2_: 718.387, Found: 718.399.

#### Bis{N-[4–(7-methoxypyrrolo[1,2-a]quinoxalin-4-yl)benzyl]-3-aminopropyl}piperazine (1e)

Pale-yellow crystals, Yield: 93%, mp =51–53 °C; ^1^H NMR δ (300 MHz, CDCl_3_) 7.97 (d, 4H, *J* = 8.20 Hz, 2 H-2′ and 2 H-6′), 7.93 (dd, 2H, *J*= 2.75 and 1.25 Hz, 2 H-1), 7.79 (d, 2H, *J* = 9.00 Hz, 2 H-9), 7.52 (d, 2H, *J* = 2.70 Hz, 2 H-6), 7.49 (d, 4H, *J* = 8.20 Hz, 2 H-3′ and 2 H-5′), 7.13 (dd, 2H, *J*= 9.00 and 2.70 Hz, 2 H-8), 6.98 (dd, 2H, *J* = 3.90 and 1.25 Hz, 2 H-3), 6.87 (dd, 2H, *J* = 3.90 and 2.75 Hz, 2 H-2), 3.93 (s, 6H, 2 CH_3_O), 3.89 (s, 4H, 2 NCH_2_), 2.71 (t, 4H, *J* = 6.90 Hz, 2 NCH_2_), 2.60–2.35 (m, 8H, 4 NCH_2_ pip.), 2.44 (t, 4H, *J* = 6.90 Hz, 2 NCH_2_), 1.76 (qt, 4H, *J* = 6.90 Hz, 2 CH_2_). ^13^C NMR δ (100 MHz, CDCl_3_) 158.5 (C-7), 155.8 (C-4), 142.8 (C-5a), 138.8 (C-3a), 138.7 (C-4′), 130.1 (C-2′ and C-6′), 129.8 (C-3′ and C-5′), 126.4 (C-1′), 122.8 (C-9a), 118.0 (C-9), 116.0 (C-8), 115.7 (C-1), 115.1 (C-2), 112.7 (C-3), 109.7 (C-6), 58.5 (NCH_2_), 57.1 (OCH_3_), 54.8 (NCH_2_), 54.6 (NCH_2_), 49.5 (NCH_2_), 27.7 (CH_2_). MALDI-TOF MS m/z [M + H]^+ ^Calcd for C_48_H_53_N_8_O_2_: 773.429, Found: 773.428.

#### Bis{N-[4–(8-methoxypyrrolo[1,2-a]quinoxalin-4-yl)benzyl]-3-aminopropyl}methylamine (1f)

Yellow crystals, Yield: 71%, mp =71–73 °C; ^1^H NMR δ (300 MHz, CDCl_3_) 7.95 (d, 2H, *J* = 9.00 Hz, 2 H-6), 7.93 (d, 4H, *J* = 8.10 Hz, 2 H-2′ and 2 H-6′), 7.86 (dd, 2H, *J* = 2.70 and 1.20 Hz, 2 H-1), 7.47 (d, 4H, *J* = 8.10 Hz, 2 H-3′ and 2 H-5′), 7.26 (d, 2H, *J* = 2.70 Hz, 2 H-9), 7.04 (dd, 2H, *J* = 9.00 and 2.70 Hz, 2 H-7), 6.95 (dd, 2H, *J* = 3.90 and 1.20 Hz, 2 H-3), 6.87 (dd, 2H, *J* = 3.90 and 2.70 Hz, 2 H-2), 3.96 (s, 6H, 2 CH_3_O), 3.88 (s, 4H, 2 NCH_2_), 2.71 (t, 4H, *J* = 6.90 Hz, 2 NCH_2_), 2.43 (t, 4H, *J* = 6.90 Hz, 2 NCH_2_), 2.25 (s, 3H, NCH_3_), 1.73 (qt, 4H, *J* = 6.90 Hz, 2 CH_2_). ^13^C NMR δ (100 MHz, CDCl_3_) 160.4 (C-8), 153.0 (C-4), 143.3 (C-3a), 138.6 (C-5a), 132.7 (C-6), 132.0 (C-4′), 130.0 (C-2′ and C-6′), 129.6 (C-3′ and C-5′), 126.2 (C-1′), 126.6 (C-9a), 115.4 (C-7), 115.3 (C-1), 114.1 (C-2), 109.5 (C-3), 98.8 (C-9), 57.6 (NCH_2_), 57.1 (OCH_3_), 55.2 (NCH_2_), 49.3 (NCH_2_), 43.7 (NCH_3_), 29.0 (CH_2_). MALDI-TOF MS m/z [M + H]^+ ^Calcd for C_45_H_48_N_7_O_2_: 718.387, Found: 718.399.

#### Bis{N-[4–(8-methoxypyrrolo[1,2-a]quinoxalin-4-yl)benzyl]-3-aminopropyl}piperazine (1 g)

Yellow oil, Yield: 97%; ^1^H NMR δ (300 MHz, CDCl_3_) 7.96 (d, 2H, *J* = 9.00 Hz, 2 H-6), 7.94 (d, 4H, *J* = 8.10 Hz, 2 H-2′ and 2 H-6′), 7.89 (dd, 2H, *J* = 2.75 and 1.20 Hz, 2 H-1), 7.47 (d, 4H, *J* = 8.10 Hz, 2 H-3′ and 2 H-5′), 7.29 (d, 2H, *J* = 2.70 Hz, 2 H-9), 7.06 (dd, 2H, *J* = 9.00 and 2.70 Hz, 2 H-7), 6.97 (dd, 2H, *J* = 3.90 and 1.20 Hz, 2 H-3), 6.89 (dd, 2H, *J* = 3.90 and 2.75 Hz, 2 H-2), 3.97 (s, 6H, 2 CH_3_O), 3.88 (s, 4H, 2 NCH_2_), 2.71 (t, 4H, *J* = 6.90 Hz, 2 NCH_2_), 2.57–2.34 (m, 8H, 4 NCH_2_ pip.), 2.43 (t, 4H, *J* = 6.90 Hz, 2 NCH_2_), 1.74 (qt, 4H, *J* = 6.90 Hz, 2 CH_2_). ^13^C NMR δ (100 MHz, CDCl_3_) 160.4 (C-8), 153.0 (C-4), 143.3 (C-3a), 138.6 (C-5a), 132.7 (C-6), 132.0 (C-4′), 130.0 (C-2′ and C-6′), 129.6 (C-3′ and C-5′), 126.2 (C-1′), 126.7 (C-9a), 115.5 (C-7), 115.4 (C-1), 114.2 (C-2), 109.5 (C-3), 98.9 (C-9), 58.4 (NCH_2_), 57.2 (OCH_3_), 55.1 (NCH_2_), 54.7 (NCH_2_), 49.4 (NCH_2_), 28.3 (CH_2_). MALDI-TOF MS m/z [M + H]^+^Calcd for C_48_H_53_N_8_O_2_: 773.429, Found: 773.446.

#### Bis{N-[4–(9-methoxypyrrolo[1,2-a]quinoxalin-4-yl)benzyl]-3-aminopropyl}methylamine (1 h)

Orange-pale oil, Yield: 92%; ^1^H NMR δ (300 MHz, CDCl_3_) 8.83 (dd, 2H, *J* = 2.70 and 1.35 Hz, 2 H-1), 7.95 (d, 4H, *J* = 8.10 Hz, 2 H-2′ and 2 H-6′), 7.66 (dd, 2H, *J* = 8.10 and 1.20 Hz, 2 H-8), 7.48 (d, 4H, *J* = 8.10 Hz, 2 H-3′ and 2 H-5′), 7.37 (t, 2H, *J* = 8.10 Hz, 2 H-7), 7.04 (dd, 2H, *J* = 8.10 and 1.20 Hz, 2 H-6), 7.00 (dd, 2H, *J* = 4.10 and 1.35 Hz, 2 H-3), 6.82 (dd, 2H, *J* = 4.10 and 2.70 Hz, 2 H-2), 4.08 (s, 6H, 2 CH_3_O), 3.89 (s, 4H, 2 NCH_2_), 2.71 (t, 4H, *J* = 6.90 Hz, 2 NCH_2_), 2.43 (t, 4H, *J* = 6.90 Hz, 2 NCH_2_), 2.25 (s, 3H, NCH_3_), 1.73 (qt, 4H, *J* = 6.90 Hz, 2 CH_2_). ^13^C NMR δ (100 MHz, CDCl_3_) 155.9 (C-9), 151.1 (C-4), 143.5 (C-3a), 139.8 (C-5a), 138.5 (C-4′), 130.1 (C-2′ and C-6′), 129.6 (C-3′ and C-5′), 127.3 (C-9a), 125.7 (C-7), 123.9 (C-6), 123.7 (C-1), 119.8 (C-1′), 114.3 (C-2), 110.2 (C-8), 109.4 (C-3), 57.6 (OCH_3_), 57.5 (NCH_2_), 55.2 (NCH_2_), 49.3 (NCH_2_), 43.7 (NCH_3_), 28.9 (CH_2_). MALDI-TOF MS m/z [M + H]^+ ^Calcd for C_45_H_48_N_7_O_2_: 718.387, Found: 718.416.

#### Bis{N-[4–(9-methoxypyrrolo[1,2-a]quinoxalin-4-yl)benzyl]-3-aminopropyl}piperazine (1i)

Pale-yellow crystals, Yield: 94%, mp =66–68 °C; ^1^H NMR δ (300 MHz, CDCl_3_) 8.84 (dd, 2H, *J* = 2.70 and 1.20 Hz, 2 H-1), 7.96 (d, 4H, *J* = 8.10 Hz, 2 H-2′ and 2 H-6′), 7.67 (dd, 2H, *J* = 8.10 and 1.20 Hz, 2 H-8), 7.48 (d, 4H, *J* = 8.10 Hz, 2 H-3′ and 2 H-5′), 7.37 (t, 2H, *J* = 8.10 Hz, 2 H-7), 7.05 (dd, 2H, *J* = 8.10 and 1.20 Hz, 2 H-6), 7.00 (dd, 2H, *J* = 4.00 and 1.20 Hz, 2 H-3), 6.85 (dd, 2H, *J* = 4.00 and 2.70 Hz, 2 H-2), 4.09 (s, 6H, 2 CH_3_O), 3.89 (s, 4H, 2 NCH_2_), 2.71 (t, 4H, *J* = 6.90 Hz, 2 NCH_2_), 2.57–2.31 (m, 8H, 4 NCH_2_ pip.), 2.43 (t, 4H, *J* = 6.90 Hz, 2 NCH_2_), 1.74 (qt, 4H, *J* = 6.90 Hz, 2 CH_2_). ^13^C NMR δ (100 MHz, CDCl_3_) 155.9 (C-9), 151.1 (C-4), 143.5 (C-3a), 139.8 (C-5a), 138.5 (C-4′), 130.1 (C-2′ and C-6′), 129.6 (C-3′ and C-5′), 127.3 (C-9a), 125.7 (C-7), 123.7 (C-6), 123.5 (C-1), 119.8 (C-1′), 114.3 (C-2), 110.2 (C-8), 109.4 (C-3), 58.4 (NCH_2_), 57.6 (OCH_3_), 55.1 (NCH_2_), 54.7 (NCH_2_), 49.4 (NCH_2_), 28.3 (CH_2_). MALDI-TOF MS m/z [M + H]^+ ^Calcd for C_48_H_53_N_8_O_2_: 773.429, Found: 773.446.

#### Bis{N-[4–(3-ethoxypyrrolo[1,2-a]quinoxalin-4-yl)benzyl]-3-aminopropyl}methylamine (1j)

Yellow oil, Yield: 85%; ^1^H NMR δ (300 MHz, CDCl_3_) 7.91 (dd, 2H, *J* = 7.80 and 1.50 Hz, 2 H-6), 7.79–7.74 (m, 8H, 2 H-2′, 2 H-6′, 2 H-1 and 2 H-9), 7.43–7.34 (m, 4H, 2 H-3′, 2 H-5′, 2 H-7 and 2 H-8), 6.48 (d, 2H, *J* = 3.00 Hz, 2 H-2), 3.92 (q, 4H, *J* = 6.90 Hz, 2 OCH_2_), 3.89 (s, 4H, 2 NCH_2_), 2.68 (t, 4H, *J* = 6.90 Hz, 2 NCH_2_), 2.43 (t, 4H, *J* = 6.90 Hz, 2 NCH_2_), 2.24 (s, 3H, NCH_3_), 1.72 (qt, 4H, *J* = 6.90 Hz, 2 CH_2_), 1.16 (t, 6H, *J* = 6.90 Hz, 2 CH_3_). ^13^C NMR δ (100 MHz, CDCl_3_) 156.2(C-4), 145.7 (C-3a), 143.6 (C-5a), 138.2 (C-4′), 137.5 (C-1′), 131.1 (C-6), 130.9 (C-2′ and C-6′), 128.5 (C-3′ and C-5′), 128.3 (C-8), 128.0 (C-9a), 126.4 (C-7), 114.0 (C-1), 113.4 (C-3),113.1 (C-9), 102.2 (C-2), 68.7 (OCH_2_), 57.6 (NCH_2_), 55.2 (NCH_2_), 49.0 (NCH_2_), 43.6 (NCH_3_), 28.8 (CH_2_), 16.1 (CH_3_). MALDI-TOF MS m/z [M + H]^+ ^Calcd for C_47_H_52_N_7_O_2_: 746.418, Found: 746.472.

#### Bis{N-[4–(3-ethoxypyrrolo[1,2-a]quinoxalin-4-yl)benzyl]-3-aminopropyl}piperazine (1k)

Yellow oil, Yield: 76%; ^1^H NMR δ (300 MHz, CDCl_3_) 7.93 (dd, 2H, *J* = 7.80 and 1.50 Hz, 2 H-6), 7.80–7.75 (m, 8H, 2 H-2′, 2 H-6′, 2 H-1 and 2 H-9), 7.47 (ddd, 2H, *J* = 7.80, 7.80 and 1.50 Hz, 2 H-7), 7.42 (d, 4H, *J* = 8.40 Hz, 2 H-3′ and 2 H-5′), 7.36 (ddd, 2H, *J* = 7.80, 7.80 and 1.50 Hz, 2 H-8), 6.51 (d, 2H, *J* = 3.00 Hz, 2 H-2), 3.94 (q, 4H, *J* = 6.90 Hz, 2 OCH_2_), 3.88 (s, 4H, 2 NCH_2_), 2.68 (t, 4H, *J* = 6.90 Hz, 2 NCH_2_), 2.56–2.31 (m, 8H, 4 NCH_2_ pip.), 2.42 (t, 4H, *J* = 6.90 Hz, 2 NCH_2_), 1.73 (qt, 4H, *J* = 6.90 Hz, 2 CH_2_), 1.17 (t, 6H, *J* = 6.90 Hz, 2 CH_3_). ^13^C NMR δ (100 MHz, CDCl_3_) 156.2 (C-4), 145.7 (C-3a), 142.6 (C-5a), 138.2 (C-4′), 137.5 (C-1′), 131.2 (C-6), 130.9 (C-2′ and C-6′), 128.5 (C-3′ and C-5′), 128.4 (C-8), 128.0 (C-9a), 126.4 (C-7), 114.0 (C-1), 113.5 (C-3), 113.2 (C-9), 102.3 (C-2), 68.8 (OCH_2_), 58.4 (NCH_2_), 55.1 (NCH_2_), 54.7 (NCH_2_), 49.1 (NCH_2_), 28.3 (CH_2_), 16.1 (CH_3_). MALDI-TOF MS m/z [M + H]^+ ^Calcd for C_50_H_57_N_8_O_2_: 801.460, Found: 801.505.

#### Bis{N-[4–(2-phenylpyrrolo[1,2-a]quinoxalin-4-yl)benzyl]-3-aminopropyl}piperazine (1 l)

Pale-yellow crystals, Yield: 79%, mp =81–83 °C; ^1^H NMR δ (300 MHz, CDCl_3_) 8.27 (d, 2H, *J* = 1.20 Hz, 2 H-1), 8.07 (dd, 2H, *J* = 7.80 and 1.30 Hz, 2 H-6), 8.03 (d, 4H, *J* = 8.10 Hz, 2 H-2′ and 2 H-6′), 7.94 (dd, 2H, *J* = 7.80 and 1.30 Hz, 2 H-9),7.71 (d, 4H, *J* = 7.50 Hz, 2 H-2” and 2 H-6”), 7.58–7.41 (m, 8H, 2 H-3′, 2 H-5′, 2 H-7 and 2 H-8), 7.45 (t, 4H, *J* = 7.80 Hz, 2 H-3” and 2 H-5”),7.35–7.30 (m, 2H, 2 H-4”), 7.26 (d, 2H, *J* = 1.20 Hz, 2 H-3), 2.73(t, 4H, *J* = 6.90 Hz, 2 NCH_2_), 2.65–2.38 (m, 8H, 4 NCH_2_ pip.), 2.45 (t, 4H, *J* = 6.90 Hz, 2 NCH_2_), 1.76 (qt, 4H, *J* = 6.90 Hz, 2 CH_2_). ^13^C NMR δ (100 MHz, CDCl_3_) 154.6 (C-4), 146.6 (C-3a), 140.0 (C-5a), 137.6 (C-4′), 135.4 (C-1”), 131.7 (C-8), 131.2 (C-1′), 130.6 (C-3” and C-5”), 130.4 (C-2′ and C-6′), 129.7 (C-7), 129.2 (C-4”), 128.6 (C-3” and C-5”), 128.1 (C-9a), 127.6 (C-3′ and C-5′), 126.9 (C-6), 118.7 (C-2), 115.0 (C-9), 112.8 (C-1), 107.2 (C-3), 58.5 (NCH_2_), 54.0 (NCH_2_), 53.2 (NCH_2_), 49.5 (NCH_3_), 31.1 (CH_2_). MALDI-TOF MS m/z [M + H]^+ ^Calcd for C_58_H_57_N_8_: 865.470, Found: 865.454.

#### Bis{N-[3-(pyrrolo[1,2-a]quinoxalin-4-yl)benzyl]-3-aminopropyl}methylamine (1m)

Pale-yellow oil, Yield: 83%; ^1^H NMR δ (300 MHz, CDCl_3_) 8.04 (dd, *J* = 7.80 and 1.80 Hz, 2 H-6), 7.99 (dd, 2H, *J* = 2.70 and 1.20 Hz, 2 H-1), 7.94 (t, 2H, *J* = 1.50 Hz, 2 H-2′), 7.89–7.85 (m, 4H, 2 H-6′ and 2 H-9), 7.55–7.42 (m, 8H, 2 H-4′, 2 H-5′, 2 H-7 and 2 H-8), 6.99 (dd, 2H, *J* = 3.90 and 1.20 Hz, 2 H-3), 6.88 (dd, 2H, *J* = 3.90 and 2.70 Hz, 2 H-2), 3.88 (s, 4H, 2 NCH_2_), 2.70 (t, 4H, *J* = 6.90 Hz, 2 NCH_2_), 2.40 (t, 4H, *J* = 6.90 Hz, 2 NCH_2_), 2.22 (s, 3H, NCH_3_), 1.72 (qt, 4H, *J* = 6.90 Hz, 2 CH_2_). ^13^C NMR δ (100 MHz, CDCl_3_) 155.8 (C-4), 142.2 (C-3a), 139.8 (C-5a), 137.6 (C-3′), 131.5 (C-8), 131.0 (C-7), 130.0 (C-2′), 129.7 (C-5′), 128.8 (C-4′),128.6 (C-6′), 128.5 (C-1′), 126.7 (C-9a), 126.6 (C-6), 116.0 (C-1), 115.4 (C-2), 115.0 (C-9), 110.1 (C-3), 57.4 (NCH_2_), 55.3 (NCH_2_), 49.4 (NCH_2_), 43.6 (NCH_3_), 28.8 (CH_2_).MALDI-TOF MS m/z [M + H]^+ ^Calcd for C_43_H_44_N_7_: 658.366, Found: 658.395.

#### Bis{N-[3-(pyrrolo[1,2-a]quinoxalin-4-yl)benzyl]-3-aminopropyl}piperazine (1n)

Pale-yellow oil, Yield: 96%; ^1^H NMR δ (300 MHz, CDCl_3_) 8.05 (dd, *J* = 7.80 and 1.80 Hz, 2 H-6), 8.01 (dd, 2H, *J* = 2.70 and 1.20 Hz, 2 H-1), 7.96 (t, 2H, *J* = 1.50 Hz, 2 H-2′), 7.92–7.86 (m, 4H, 2 H-6′ and 2 H-9), 7.56–7.43 (m, 8H, 2 H-4′, 2 H-5′, 2 H-7 and 2 H-8), 7.00 (dd, 2H, *J* = 3.90 and 1.20 Hz, 2 H-3), 6.90 (dd, 2H, *J* = 3.90 and 2.70 Hz, 2 H-2), 3.91 (s, 4H, 2 NCH_2_), 2.69 (t, 4H, *J* = 6.90 Hz, 2 NCH_2_), 2.58–2.34 (m, 8H, 4 NCH_2_ pip.), 2.34 (t, 4H, *J* = 6.90 Hz, 2 NCH_2_), 1.95 (bs, 2H, 2 NH), 1.68 (qt, 4H, *J* = 6.90 Hz, 2 CH_2_). ^13^C NMR δ (100 MHz, CDCl_3_) 155.8 (C-4), 142.2 (C-3a), 139.9 (C-5a), 137.6 (C-3′), 131.5 (C-8), 131.0 (C-7), 130.0 (C-2′), 129.7 (C-5′), 128.9 (C-4′), 128.6 (C-6′), 128.5 (C-1′), 126.8 (C-9a), 126.7 (C-6), 116.0 (C-1), 115.4 (C-2), 115.0 (C-9), 110.1 (C-3), 58.4 (NCH_2_), 55.2 (NCH_2_), 54.6 (NCH_2_), 49.5 (NCH_2_), 28.2 (CH_2_). MALDI-TOF MS m/z [M + H]^+ ^Calcd for C_46_H_49_N_8_: 713.408, Found: 713.447.

#### Bis{N-[3–(8-methoxypyrrolo[1,2-a]quinoxalin-4-yl)benzyl]-3-aminopropyl}methylamine (1o)

Yellow oil, Yield: 97%; ^1^H NMR δ (300 MHz, CDCl_3_) 7.95 (d, 2H, *J* = 8.70 Hz, 2 H-6), 7.90 (dd, 2H, *J* = 1.50 and 1.50 Hz, 2 H-2′), 7.87–7.83 (m, 2H, 2 H-4′), 7.85 (dd, 2H, *J* = 2.70 and 1.20 Hz, 2 H-1), 7.47–7.43 (m, 4H, 2 H-5′ and 2 H-5′), 7.25 (d, 2H, *J* = 2.70 Hz, 2 H-9), 7.03 (dd, 2H, *J* = 8.85 and 2.70 Hz, 2 H-7), 6.94 (dd, 2H, *J* = 3.90 and 1.20 Hz, 2 H-3), 6.86 (dd, 2H, *J* = 3.90 and 2.70 Hz, 2 H-2), 3.94 (s, 6H, 2 CH_3_O),3.86 (s, 4H, 2 NCH_2_), 2.68 (t, 4H, *J* = 6.90 Hz, 2 NCH_2_), 2.39 (t, 4H, *J* = 6.90 Hz, 2 NCH_2_), 2.20 (s, 3H, NCH_3_), 1.69 (qt, 4H, *J* = 6.90 Hz, 2 CH_2_). ^13^C NMR δ (100 MHz, CDCl_3_) 160.4 (C-8), 153.1 (C-4), 141.8 (C-3a), 140.0 (C-5a), 132.7 (C-6), 131.9 (C-3′), 130.8 (C-2′), 129.9 (C-5′), 129.7 (C-4′), 129.2 (C-1′), 128.6 (C-6′), 126.6 (C-9a), 115.5 (C-7), 115.4 (C-1), 114.2 (C-2), 109.5 (C-3), 98.8 (C-9), 57.5 (NCH_2_), 57.1 (OCH_3_), 55.2 (NCH_2_), 49.3 (NCH_2_), 43.6 (NCH_3_), 28.6 (CH_2_). MALDI-TOF MS m/z [M + H]^+ ^Calcd for C_45_H_48_N_7_O_2_: 718.387, Found: 718.433.

#### Bis{N-[3–(8-methoxypyrrolo[1,2-a]quinoxalin-4-yl)benzyl]-3-aminopropyl}piperazine (1p)

Yellow oil, Yield: 75%; ^1^H NMR δ (300 MHz, CDCl_3_) 7.97 (d, 2H, *J* = 9.00 Hz, 2 H-6), 7.93 (dd, 2H, *J* = 1.50 and 1.50 Hz, 2 H-2′), 7.88 (dd, 2H, *J* = 2.70 and 1.20 Hz, 2 H-1),7.88–7.84 (m, 2H, 2 H-4′), 7.50–7.46 (m, 4H, 2 H-5′ and 2 H-5′), 7.28 (d, 2H, *J* = 2.70 Hz, 2 H-9), 7.06 (dd, 2H, *J* = 9.00 and 2.70 Hz, 2 H-7), 6.96 (dd, 2H, *J* = 4.00 and 1.20 Hz, 2 H-3), 6.88 (dd, 2H, *J* = 4.00 and 2.70 Hz, 2 H-2), 3.96 (s, 6H, 2 CH_3_O),3.90 (s, 4H, 2 NCH_2_), 2.69 (t, 4H, *J* = 6.90 Hz, 2 NCH_2_), 2.53–2.34 (m, 8H, 4 NCH_2_ pip.), 2.33 (t, 4H, *J* = 6.90 Hz, 2 NCH_2_), 2.20 (bs, 2H, 2 NH), 1.68 (qt, 4H, *J* = 6.90 Hz, 2 CH_2_). ^13^C NMR δ (100 MHz, CDCl_3_) 160.5 (C-8), 153.2 (C-4), 142.0 (C-3a), 140.0 (C-5a), 132.7 (C-6), 132.0 (C-3′), 130.7 (C-2′), 130.0 (C-5′), 129.6 (C-4′), 129.2 (C-1′), 128.6 (C-6′), 126.7 (C-9a), 115.5 (C-7), 115.4 (C-1), 114.2 (C-2), 109.5 (C-3), 98.9 (C-9), 58.4 (NCH_2_), 57.2 (OCH_3_), 55.2 (NCH_2_), 54.6 (NCH_2_), 49.5 (NCH_2_), 28.1 (CH_2_). MALDI-TOF MS m/z [M + H]^+^Calcd for C_48_H_53_N_8_O_2_: 773.429, Found: 773.464.

#### Bis{N-[4-(indolo[1,2-a]quinoxalin-6-yl)benzyl]-3-aminopropyl}piperazine (1q)

Yellow oil, Yield: 86%; ^1^H NMR δ (300 MHz, CDCl_3_) 8.55 (dd, 2H, *J* = 8.10 and 1.20 Hz, 2 H-11), 8.51 (dd, 2H, *J* = 8.70 and 0.90 Hz, 2 H-1), 8.09 (dd, 2H, *J* = 8.00 and 1.65 Hz, 2 H-4), 8.00 (d, 4H, *J* = 8.40 Hz, 2 H-2′ and 2 H-6′), 7.95 (d, 2H, *J* = 7.80 Hz, 2 H-8), 7.67–7.60 (m, 4H,2 H-2 and 2 H-3), 7.53 (d, 4H, *J* = 8.40 Hz, 2 H-3′ and 2 H-5′), 7.48–7.43 (m, 4H, 2 H-9 and 2 H-10), 7.27 (s, 2H, 2 H-7), 3.92 (s, 4H, 2 NCH_2_), 2.75 (t, 4H, *J* = 6.90 Hz, 2 NCH_2_), 2.63–2.41 (m, 8H, 4 NCH_2_ pip.), 2.46 (t, 4H, *J* = 6.90 Hz, 2 NCH_2_), 1.96 (bs, 2H, 2 NH), 1.77 (qt, 4H, *J* = 6.90 Hz, 2 CH_2_). ^13^C NMR δ (100 MHz, CDCl_3_) 157.3 (C-6), 142.8 (C-6a), 138.6 (C-11a), 137.7 (C-4a), 134.4 (C-4′), 131.9 (C-3), 131.6 (C-1′), 130.5 (C-8a), 130.2 (C-2′ and C-6′), 129.9 (C-3′ and C-5′), 129.7 (C-2), 125.8 (C-4), 125.6 (C-1), 124.1 (C-9), 124.0 (C-10), 116.0 (C-8 and C-11), 103.8 (C-7), 58.5 (NCH_2_), 54.6 (NCH_2_), 49.5 (NCH_2_), 27.6 (CH_2_). MALDI-TOF MS m/z [M + H]^+ ^Calcd for C_54_H_53_N_8_: 813.439, Found: 813.432.

#### Bis{N-[(4-phenylpyrrolo[1,2-a]quinoxalin-2-yl)methyl]-3-aminopropyl}piperazine (1r)

Pale-yellow crystals, Yield: 70%, mp =62–64 °C; ^1^H NMR δ (300 MHz, CDCl_3_) 8.04 (dd, 2H, *J* = 7.80 and 1.50 Hz, 2 H-6), 8.03–7.97 (m, 4H, 2 2 H-2′ and 2 H-6′), 7.98 (d, 2H, *J* = 1.10 Hz, 2 H-1), 7.85 (dd, 2H, *J* = 7.80 and 1.50 Hz, 2 H-9), 7.57–7.53 (m, 8H, 2 H-3′, 2 H-4′, 2 H-5′ and 2 H-7), 7.47 (ddd, 2H, *J* = 7.80, 7.80, 1.50 Hz, 2 H-8), 6.94 (d, 2H, *J* = 1.10 Hz, 2 H-3), 3.95 (s, 4H, 2 NCH_2_), 2.75 (t, 4H, *J* = 6.90 Hz, 2 NCH_2_), 2.61–2.35 (m, 8H, 4 NCH_2_ pip.), 2.40 (t, 4H, *J* = 6.90 Hz, 2 NCH_2_), 1.93 (bs, 2H, 2 NH), 1.72 (qt, 4H, *J* = 6.90 Hz, 2 CH_2_). ^13^C NMR δ (100 MHz, CDCl_3_) 155.2 (C-4), 139.5 (C-3a), 137.5 (C-5a), 131.6 (C-7), 131.4 (C-8), 130.1 (C-3′ and C-5′), 130.0 (C-2′ and C-6′), 129.2 (C-6), 128.0 (C-1′), 127.0 (C-9a), 126.7 (C-4′), 115.3 (C-9), 114.2 (C-2), 109.9 (C-1), 109.8 (C-3), 58.2 (NCH_2_), 54.3 (NCH_2_), 54.1 (NCH_2_), 49.3 (NCH_2_), 46.4 (NCH_2_), 24.8 (CH_2_). MALDI-TOF MS m/z [M + H]^+ ^Calcd for C_46_H_49_N_8_: 713.408, Found: 713.412.

#### Tris{N-[4–(7-methoxypyrrolo[1,2-a]quinoxalin-4-yl)benzyl]-3-aminopropyl}amine (1s)

Yellow oil, Yield: 94%; ^1^H NMR δ (300 MHz, CDCl_3_) 7.94 (d, 6H, *J* = 8.40 Hz, 3 H-2′ and 3 H-6′), 7.87 (dd, 3H, *J* = 2.70 and 1.35 Hz, 3 H-1), 7.74 (d, 3H, *J* = 9.00 Hz, 3 H-9), 7.48 (d, 6H, *J* = 8.40 Hz, 3 H-3′ and 3 H-5′), 7.47 (d, 3H, *J* = 2.70 Hz, 3 H-6), 7.09 (dd, 3H, *J* = 9.00 and 2.70 Hz, 3 H-8), 6.93 (dd, 3H, *J* = 4.05 and 1.35 Hz, 3 H-3), 6.81 (dd, 3H, *J*= 4.05 and 2.70 Hz, 3 H-2), 3.90 (s, 9H, 3 CH_3_O), 3.88 (s, 6H, 3 NCH_2_), 2.70 (t, 6H, *J* = 6.90 Hz, 3 CH_2_), 2.52 (t, 6H, *J* = 6.90 Hz, 3 CH_2_), 1.71 (qt, 6H, *J* = 6.90 Hz, 6 CH_2_). ^13^C NMR δ (100 MHz, CDCl_3_) 158.5 (C-7), 155.8 (C-4), 143.1 (C-5a), 138.76 (C-3a and C-4′), 130.1 (C-2′ and C-6′), 129.8 (C-3′ and C-5′), 126.4 (C-1′), 122.8 (C-9a), 117.9 (C-9), 115.9 (C-8), 115.6 (C-1), 115.0 (C-2), 112.6 (C-3), 109.7 (C-6), 57.1 (OCH_3_), 55.1 (NCH_2_), 53.7 (NCH_2_), 49.2 (NCH_2_), 28.4 (CH_2_). MALDI-TOF MS m/z [M + H]^+ ^Calcd for C_66_H_67_N_10_O_3_: 1047.540, Found: 1047.613.

#### Tris{N-[3–(8-methoxypyrrolo[1,2-a]quinoxalin-4-yl)benzyl]-3-aminopropyl}amine (1t)

Orange oil, Yield: 79%; ^1^H NMR δ (300 MHz, CDCl_3_) 7.94 (d, 3H, *J* = 9.00 Hz, 3 H-6), 7.89 (dd, 3H, *J* = 1.50 and 1.50 Hz, 3 H-2′), 7.86–7.80 (m, 6H, 3 H-4′and 3 H-1), 7.45–7.42 (m, 6H, 3 H-6′ and 3 H-5′), 7.23 (d, 3H, *J* = 2.70 Hz, 3 H-9), 7.03 (dd, 3H, *J* = 9.00 and 2.70 Hz, 3 H-7), 6.93 (dd, 3H, *J* = 3.90 and 1.20 Hz, 3 H-3), 6.85 (dd, 3H, *J* = 3.90 and 2.70 Hz, 3 H-2), 3.96 (s, 9H, 3 CH_3_O), 3.83 (s, 6H, 3NCH_2_), 2.65 (t, 6H, *J* = 6.90 Hz, 3 NCH_2_), 2.47 (t, 6H, *J* = 6.90 Hz, 3 NCH_2_), 1.63 (qt, 6H, *J* = 6.90 Hz, 3 CH_2_). ^13^C NMR δ (100 MHz, CDCl_3_) 160.4 (C-8), 153.1 (C-4), 141.9 (C-3a), 140.0 (C-5a), 132.7 (C-6), 132.0 (C-3′), 130.8 (C-2′), 129.9 (C-5′), 129.7 (C-4′), 129.2 (C-1′), 128.6 (C-6′), 126.6 (C-9a), 115.4 (C-7), 115.3 (C-1), 114.1 (C-2), 109.5 (C-3), 98.7 (C-9), 57.1 (OCH_3_), 55.3 (NCH_2_), 53.7 (NCH_2_), 49.3 (NCH_2_), 28.4 (CH_2_). MALDI-TOF MS m/z [M + H]^+ ^Calcd for C_66_H_67_N_10_O_3_: 1047.540, Found: 1047.598.

#### N,N′-[oxybis(2,1-ethanediyloxy-2,1-ethanediyl)]bis-3–(8-methoxypyrrolo[1,2-a]quinoxalin-4-yl)benzylamine (1u)

Yellow oil, Yield: 92%; ^1^H NMR δ (300 MHz, CDCl_3_) 7.95 (d, 3H, *J* = 9.00 Hz, 2 H-6), 7.93 (dd, 3H, *J* = 1.50 and 1.50 Hz, 2 H-2′), 7.88–7.83 (m, 4H, 2 H-4′and 2 H-1), 7.50–7.45 (m, 4H, 2 H-6′ and 2 H-5′), 7.26 (d, 2H, *J* = 2.70 Hz, 2 H-9), 7.05 (dd, 2H, *J* = 9.00 and 2.70 Hz, 2 H-7), 6.95 (dd, 2H, *J* = 3.90 and 1.20 Hz, 2 H-3), 6.87 (dd, 2H, *J* = 3.90 and 2.70 Hz, 2 H-2), 3.95 (s, 6H, 2 CH_3_O), 3.91 (s, 4H, 2 NCH_2_), 3.64–3.58 (m, 12H, 6 OCH_2_), 2.83 (t, 4H, *J* = 5.40 Hz, 2 NCH_2_), 2.18 (bs, 2H, 2 NH). ^13^C NMR δ (100 MHz, CDCl_3_) 160.5 (C-8), 153.1 (C-4), 141.3 (C-3a), 140.0 (C-5a), 132.7 (C-6), 132.0 (C-3′), 131.0 (C-2′), 129.9 (C-5′ and C-4′), 129.2 (C-1′), 128.7 (C-6′), 126.7 (C-9a), 115.4 (C-7), 115.3 (C-1), 114.2 (C-2), 109.6 (C-3), 98.9 (C-9), 71.8 (OCH_2_), 71.6 (OCH_2_), 71.5 (OCH_2_), 56.7 (OCH_3_), 54.8 (NCH_2_), 49.8 (NCH_2_). MALDI-TOF MS m/z [M + H]^+ ^Calcd for C_46_H_49_N_6_O_5_: 765.376, Found: 765.457.

### In vitro antiplasmodial activity

The *in vitro* antiplasmodial activities were tested over concentrations ranging from 39 nM to 40 μM against culture-adapted *Plasmodium falciparum* reference strains 3D7 and W2. The former strain is susceptible to chloroquine (CQ) but displays a decreased susceptibility to mefloquine (MQ) while the latter is considered as resistant to chloroquine. The parasites were cultivated in RPMI medium (Sigma-Aldrich, Lyon, France) supplemented with 0.5% Albumax I (Life Technologies corporation, Paisley, United Kingdom), hypoxanthine (Sigma-Aldrich), gentamicin (Sigma-Aldrich), and human erythrocytes and were incubated at 37 °C in a candle jar, as described previously^37^. The *P. falciparum* drug susceptibility test was carried out in 96-well flat bottom sterile plates under a final volume of 250 μL. After a 48 h incubation with the drugs, quantities of DNA in treated and control cultures of parasites in human erythrocytes were compared according to the SYBR Green I (Sigma-Aldrich) fluorescence-based method^38^^,^^39^. Briefly, after incubation, plates were frozen at -20 °C until use. They were then left to thaw for 2 h at room temperature and 100 μL of the homogenized culture were transferred to 96-well flat bottom sterile black plates (Nunc Inc) already containing 100 μL of the SYBR Green I lysis buffer (2xSYBR Green, 20 mM Tris base pH 7.5, 5 mM EDTA, 0.008% w/v saponin, 0.08% w/v Triton X-100). A negative control, controls treated by solvents (DMSO and H_2_O, typically) and positive controls (chloroquine and mefloquine) were added to each set of experiments. Plates were incubated for 1 h at room temperature and then read on a fluorescence plate reader (Tecan, Austria) using excitation and emission wavelengths of 485 and 535 nm, respectively. Concentrations inhibiting 50% of the parasite′s growth (half maximal inhibitory concentration or IC_50_ values) were then calculated from the obtained experimental results using a regression program available on line^40^.

### In vitro antileishmanial activity

*L. donovani* (MHOM/IN/00/DEVI) used in this study was provided by the CNR *Leishmania* (Montpellier, France). The effects of the tested compounds on the growth of *L. donovani* (MHOM/IN/00/DEVI)promastigotes were assessed by MTT assay^41^ .Briefly, promastigotes in log-phase in Schneider′s medium supplemented with 20% fetal calf serum (FCS), 2 mM L-glutamine and antibiotics (100 U/mL penicillin and 100 μg/mL streptomycin), were incubated at an average density of 10^6^ parasites/mL in sterile 96-well plates with various concentrations of compounds dissolved in DMSO (final concentration less than 0.5% v/v), in duplicate. Appropriate controls treated by DMSO and pentamidine or amphotericin B (reference drugs purchased from Sigma-Aldrich) were added to each set of experiments. After a 72 h incubation period at 27 °C, parasite metabolic activity was determined. Each plate-well was then microscope-examined for detecting possible precipitate formation. 20 μL of MTT 3–(4,5-dimethylthiazol-2-yl)-2,5-diphenyltetrazolium bromide) solution (5 mg/mL) were added to each well followed by incubation for another 4 h. The enzyme reaction was then stopped by addition of 100 μL of 50% isopropanol – 10% sodium dodecyl sulfate^42^ .Plates were shaken vigorously (300 rpm) for 10 minutes and the absorbance measured in a plate reader at 570 nm in a BIO-TEK ELx808 Absorbance Microplate Reader. Inhibitory concentration 50% (IC_50_) was defined as the concentration of drug required to inhibit by 50% the metabolic activity of *L. donovani* promastigotes compared to the control. IC_50_ were calculated by non-linear regression analysis processed on doseresponse curves, using TableCurve 2D V5.0 software. IC_50_ values represent the mean value calculated from three independent experiments.

### Cytotoxicity evaluation

A cytotoxicity evaluation was realized according to the method of Mosmann^41^ with slight modificationsto determine the cytotoxic concentrations 50% (CC_50_) and using doxorubicin as a cytotoxic reference-compound. These assays were performed toward the human HepG2 cell line (HepG2 CC_50_). HepG2 (hepatocarcinoma cell line purchased from ATCC, ref HB-8065) is a commonly used human-derived hepatocarcinoma cell line that has shown characteristics similar to those of primary hepatocytes. These cells express many of the hepatocyte-specific metabolic enzymes, thus enabling the cytotoxicity of tested product metabolites to be evaluated. Briefly, cells in 100 μL of complete RPMI medium, [RPMI supplemented with 10% FCS, 1% l-glutamine (200 mM) and penicillin (100 U/mL)/streptomycin (100 μg/mL)] were inoculated into each well of 96-well plates and incubated at 37 °C in a humidified 6% CO_2_. After 24 h incubation, 100 μL of medium with various product concentrations dissolved in DMSO (final concentration less than 0.5% v/v) were added and the plates were incubated for 72 h at 37 °C. Duplicate assays were performed for each sample. Each plate-well was then microscope-examined for detecting possible precipitate formation before the medium was aspirated from the wells. 100 μL of MTT solution (0.5 mg/mL in medium without FCS) were then added to each well. Cells were incubated for 2 h at 37 °C. After this time, the MTT solution was removed and DMSO (100 μL) was added to dissolve the resulting blue formazan crystals. Plates were shaken vigorously (300 rpm) for 5 minutes. The absorbance was measured at 570 nm with 630 nm as reference wavelength spectrophotometer using a BIO-TEK ELx808 Absorbance Microplate Reader. DMSO was used as blank and doxorubicin (Sigma Aldrich) as positive control. Cell viability was calculated as percentage of control (cells incubated without compound). The 50% cytotoxic concentration was determined from the dose-response curve by using the TableCurve 2D V5.0 software (Systat Software, San Jose, CA).

### FRET melting experiments

FRET melting experiments were performed with dual-labeled oligonucleotides mimicking the *Plasmodium* telomeric sequences FPf1T [FAM-^5^′(GGGTTTA)_3_-GGG^3^′-TAMRA] and FPf8T [FAM-^5^′(GGGTTCA)_3_GGG^3^′-TAMRA] and the human telomeric sequence F21T [FAM-(GGGTTA)_3_-GGG^3^′- TAMRA]^36^^,^^43^. The oligonucleotides were prefolded in 10 mM lithium cacodylate buffer (pH 7.2), with 10 mM KCl and 90 mM LiCl (K^+ ^condition). The FAM emissions were recorded at 516 nm using a 492-nm excitation wavelength in the absence and presence of a single compound as a function of temperature (25 to 95 °C) in 96-well microplates by using a Stratagene MX3000P real-time PCR device at a rate of 1 °C.min^−^^1^. The data obtained were normalized between 0 and 1, and the temperature required for half-denaturationof oligonucleotides corresponded to the emission value of 0.5 was calculated as ΔT_m_. Each experiment was performed in duplicate with 200 nM of labeled oligonucleotide and 0, 1, 2, or 5 μM of compound under K^+ ^condition. For each compound, two independent experiments were carried out. The data were plotted using OriginPro 9.1 software (OriginLab Corporation, Northampton, MA).

## Results and discussion

### Chemistry

**Scheme 1.**
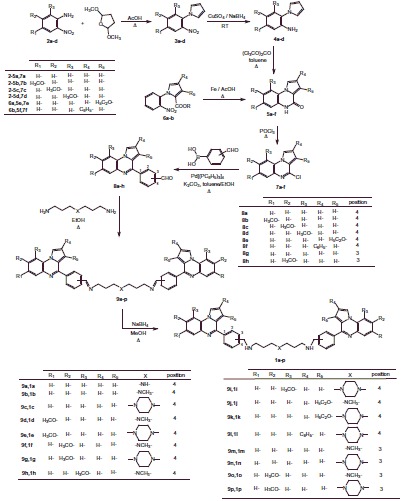
Synthesis of bispyrrolo[1,2-*a*]quinoxalines **1a–p**.

The reported bis{*N*-[(pyrrolo[1,2-*a*]quinoxalin-4-yl)benzyl]-3-aminopropyl}amine or piperazine derivatives **1a–p** were synthesized in seven steps from 2-nitroaniline (Scheme 1). Preparation of 1–(2-nitrophenyl)pyrroles **3a–d** was performed according to the Clauson-Kaas reaction run under micro-wave irradiation starting from 2-nitroaniline and 2,5-dimethoxytetrahedrofuran in acetic acid. This pathway partially involved synthetic methodologies already described by our group^20–23^^,^^27^. The resulting 1–(2-nitrophenyl)pyrrole intermediates **3a–d** was subsequently reduced into the attempted 1–(2-aminophenyl)pyrroles **4a–d** using a sodium borohydride-copper (II) sulfate in ethanol at room temperature. This NaBH_4_-CuSO_4_ system was found to be quite powerful in reducing our aromatic nitro group with excellent yield (73–85%). The reaction of **4a–d** with triphosgene in toluene gave the lactams **5a–d**^22^. Reduction of the nitro moiety of **6a–b** with iron in hot glacial acetic acid produced the spontaneous ring closure onto the ester to afford the desired the lactams **5e–f** through a one-pot reduction-cyclization step^27^^,^^25^.

The lactams **5a–f** were subsequently chlorodehydroxylated with phosphorous oxychloride, leading to the 4-chloropyrrolo[1,2-*a*]quinoxalines **7a–f**.

**Scheme 2.**
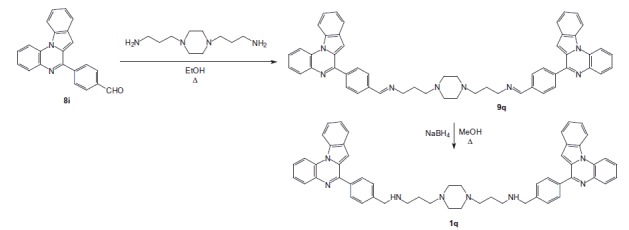
Synthesis of bisindolo[1,2-*a*]quinoxaline **1q**.

**Scheme 3.**
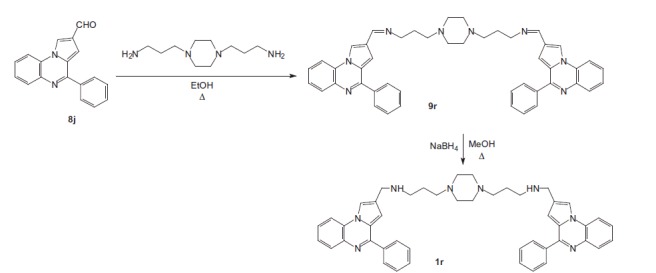
Synthesis of bispyrrolo[1,2-*a*]quinoxaline **1r**.

Coupling chloro derivatives **7a–f** with 3- or 4-formylphenylboronic acid in the presence of Pd(PPh_3_)_4_ as a catalyst under Suzuki-Miyaura cross-coupling conditions proceeded to afford the substituted benzaldehydes **8a–h**^22^^,^^27^^,^^28^^,^^30^. Reaction of primary amines, such as 3,3′-diamino-*N*-methyldipropylamine or *N*-(3-aminopropyl)-1,3-propanediamine or 1,4-bis(3-aminopropyl)piperazine), with the latter **7a–f** gave the di-imines **9a–p**, reduced into the bis{*N*-[(pyrrolo[1,2-*a*]quinoxalin-4-yl)benzyl]-3-aminopropyl}amines **1a–p** using sodium borohydride in methanol. The same pathway was used for the synthesis of bisindoloquinoxaline **1q** and bis(4-phenyl)pyrroloquinoxaline **1r** from aldehydes **8i** and **8j**, respectively (Schemes 2 and 3).

**Scheme 4.**
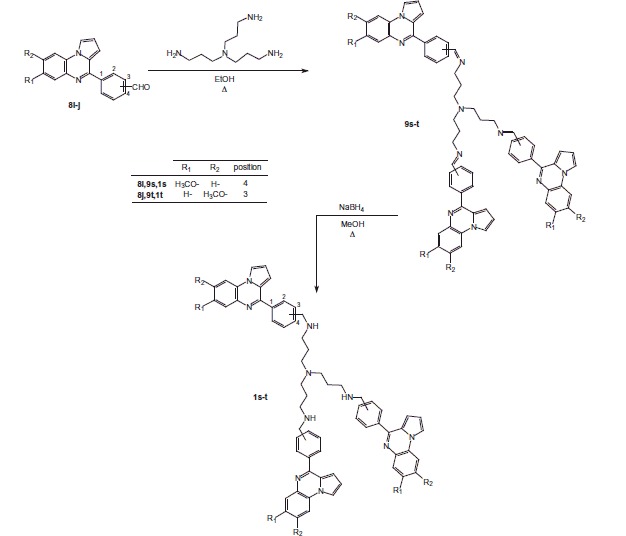
Synthesis of trispyrrolo[1,2-*a*]quinoxalines **1s–t**.

**Scheme 5.**
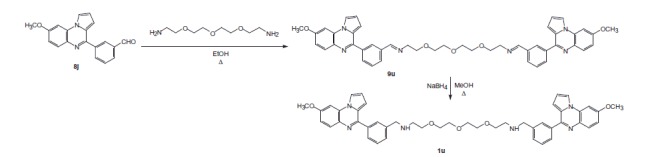
Synthesis of bispyrrolo[1,2-*a*]quinoxaline **1u**.

This strategy was also used to prepare the tris{*N*-[(pyrrolo[1,2-*a*]quinoxalin-4-yl)benzyl]-3-aminopropyl}amines **1s–t** (Scheme 4) and *N*,*N′*-[oxybis(2,1-ethanediyloxy-2,1-ethanediyl)]bis-(pyrrolo[1,2-*a*]quinoxalin-4-yl)benzylamine **1u** (Scheme 5).

All these quinoxaline compounds **1a–u** were then converted into their ammonium oxalate salts by treatment with oxalic acid in refluxing isopropanol (Table 1).

**Table 1.** Physical properties of the final amines **1a–t.**

Compound		Salt^a^	mp (°C)^b^	% Yield^c^
**1a**	Yellow crystals	3 (COOH)_2_	>260	83
**1b**	Yellow crystals	3 (COOH)_2_	245–248	79
**1c**	Yellow crystals	4 (COOH)_2_	>260	85
**1d**	Yellow crystals	3 (COOH)_2_	224–226	81
**1e**	Yellow crystals	4 (COOH)_2_	246–249	43
**1f**	Yellow crystals	3 (COOH)_2_	240–242	76
**1g**	Yellow crystals	4 (COOH)_2_	236–239	70
**1h**	Pale-orange crystals	3 (COOH)_2_	160–162	73
**1i**	Yellow crystals	4 (COOH)_2_	>260	75
**1j**	Yellow crystals	3 (COOH)_2_	192–195	88
**1k**	Yellow crystals	4 (COOH)_2_	218–220	91
**1l**	Yellow crystals	4 (COOH)_2_	239–243	56
**1m**	Yellow crystals	4 (COOH)_2_	209–211	82
**1n**	Yellow crystals	4 (COOH)_2_	220–223	68
**1o**	Yellow crystals	3 (COOH)_2_	188–191	81
**1p**	Yellow crystals	4 (COOH)_2_	234–237	86
**1q**	Oranges crystals	4 (COOH)_2_	>260	65
**1r**	Pale-yellow crystals	4 (COOH)_2_	>260	88
**1s**	Yellow crystals	4 (COOH)_2_	192–194	77
**1t**	Yellow crystals	4 (COOH)_2_	184–187	88
**1u**	Beige crystals	2 (COOH)_2_	141–144	76

a The stoichiometry and composition of the salts were determined by elemental analyses (within ±0.4% of the theoretical values).

b Crystallization solvent: 2-PrOH–H_2_O.

c The yields only included the conversions into the ammonium oxalates.

### Biological activity

#### In vitro antimalarial activity

All new substituted bis- and trispyrrolo[1,2-*a*]quinoxaline derivatives **1a–u** were evaluated for their antimalarial activity *in vitro* on *P. falciparum* CQ-resistant strain W2 (IC_50_ CQ =0.20 μM) and the CQ-sensitive and MQ decrease susceptibility strain 3D7 (IC_50_ CQ =0.08 μM). As shown in Table 2, they were found to have IC_50_ between 0.05–4.20 μM on W2, and 0.04–8.96 μM on 3D7 *P. falciparum* strains, respectively.

**Table 2.** *In vitro* sensitivity of compounds **1a-u** on *P. falciparum* and *L. donovani* strains, and cytotoxicity on the HepG2 cell line.

	*P. falciparum strains* IC_50_values (μΜ)^a^			Cytotoxicity CC_50_ values (μM)^c^	Selectivity Index^d^	
	W2	3D7	*L. donovani* IC_50_ values (μΜ)^b^	HepG2	HepG2/W2	HepG2/3D7
Chloroquine^e^	0.20 ± 0.03	0.08 ± 0.003	n.d.^g^	30	150	300
Mefloquine^e^	0.032 ± 0.001	0.08 ± 0.008	n.d.^g^	n.d.^g^	n.d.^g^	n.d.^g^
Pentamidine^f^	n.d.^g^	n.d.^g^	5.5 ± 0.8	2.3 ± 0.5	n.d.^g^	n.d.^g^
Amphotericin B^f^	n.d.^g^	n.d.^g^	0.1 ± 0.04	8.8 ± 0.6	n.d.^g^	n.d.^g^
**1a**	0.85 ± 0.15	3.42 ± 0.54	1.0 ± 0.2	1.81 ± 0.10	2.13	0.53
**1b**	4.20 ± 0.65	1.20 ± 0.20	n.d.^g^	1.56 ± 0.60	0.37	1.30
**1c**	3.40 ± 0.74	0.80 ± 0.12	1.14 ± 0.2	1.25 ± 0.30	0.37	1.56
**1d**	0.34 ± 0.04	4.14 ± 0.03	3.89 ± 0.3	2.57 ± 0.7	7.56	0.62
**1e**	0.21 ± 0.03	2.62 ± 0.25	2.52 ± 0.1	1.46 ± 0.6	6.95	0.56
**1f**	0.13 ± 0.01	5.85 ± 0.07	4.25 ± 0.2	3.38 ± 0.9	26	0.58
**1g**	0.36 ± 0.04	2.22 ± 0.25	1.07 ± 0.1	2.14 ± 1.40	5.94	0.96
**1h**	0.44 ± 0.11	0.25 ± 0.04	2.07 ± 0.02	2.44 ± 0.4	5.54	9.76
**1i**	0.32 ± 0.04	1.28 ± 0.10	1.58 ± 0.1	1.33 ± 0.3	4.16	1.04
**1j**	0.49 ± 0.16	0.26 ± 0.03	4.26 ± 0.1	1.95 ± 0.3	3.98	7.50
**1k**	0.09 ± 0.01	0.19 ± 0.01	≥10^h^	1.48 ± 0.5	16.44	7.79
**1l**	1.39 ± 0.13	8.96 ± 0.69	n.d.^g^	n.d.^g^	n.d.^g^	n.d.^g^
**1m**	0.12 ± 0.01	1.07 ± 0.16	2.80 ± 0.1	1.94 ± 1.0	16.17	1.81
**1n**	0.05 ± 0.01	0.37 ± 0.04	3.71 ± 0.4	2.03 ± 1.2	40.6	5.49
**1o**	0.206 ± 0.03	0.18 ± 0.02	3.51 ± 0.1	1.50 ± 0.9	7.28	8.33
**1p**	0.17 ± 0.03	0.04 ± 0.004	3.39 ± 0.4	1.57 ± 0.8	9.23	39.25
**1q**	n.d.^g^	1.00 ± 0.05	0.57 ± 0.1	1.56 ± 0.70	1.56	n.d.^g^
**1r**	0.75 ± 0.10	7.22 ± 0.77	n.d.^g^	n.d.^g^	n.d.^g^	n.d.^g^
**1s**	0.29 ± 0.05	0.20 ± 0.04	≥10^h^	1.66 ± 0.5	5.72	8.30
**1t**	0.19 ± 0.02	0.24 ± 0.03	≥10^h^	1.72 ± 0.6	9.05	7.17
**1u**	0.25 ± 0.08	0.076 ± 0.009	≥10^h^	1.54 ± 0.7	6.16	20.26

a IC_50_ values were measured on the chloroquine-resistant and mefloquine-sensitive strain W2 and the chloroquine-sensitive and mefloquine-resistant strain 3D7.

b IC_50_ values were measured on the promastigotes of *Leishmania donovani* strain. The IC_50_ (μM) values correspond to the mean ± standard deviation from three independent experiments.

c CC_50_ values were measured on the HepG2 cell line. The CC_50_ (μM) values correspond to the mean ± standard deviation from three independent experiments.

d Selectivity Index (SI) was defined as the ratio between the CC_50_ value on the HepG2 cells and the IC_50_ value against the *P. falciparum* W2 or 3D7 strain.

e Chloroquine and mefloquine were used as antiplasmodial drug-compounds of reference.

f Pentamidine and Amphotericin B were used as antileishmanial drug-compounds of reference.

g n.d.: not done.

h No activity noted at the higher concentration tested.

For the pyrroloquinoxalines unsubstituted on their heterocyclic moiety (compounds **1a–c**), compound **1a** joined by a bis-(3-aminopropyl)amine linker was found more active (up to 4 to 5 times) on the W2 strain than its counterparts with bis-(3-aminopropyl)methylamine (compounds **1b**) or bis-(3-aminopropyl)piperazine linkages (compounds **1c**): i.e. IC_50 _=_ _0.85 μM for **1a*** versus* 4.20 and 3.40 μM for **1b** and **1c**, respectively.As a general rule against the W2 strain, the introduction of a methoxy substituent in position 7, 8 or 9 of the bis-pyrrolo[1,2-*a*]quinoxaline skeleton linked in position 4 of the benzyl ring (compounds **1d–i**) strongly increased the antiparasitic activity in comparison with their respective unsubstituted bispyrrolo[1,2-*a*]quinoxaline homologs **1b–c**: i.e. IC_50 _=_ _0.13–0.44 μM for **1d–i** and 3.40–4.20 μM for **1b–c**. Moreover, introduction of an ethoxy substituent in position C-3 of the pyrrolo[1,2-*a*]quinoxaline moieties joined by a bis-(3-aminopropyl) piperazine linker (compound **1k**) was found more active (up to 38 times) than its non-substituted homolog **1c** (IC_50 _=_ _0.09 μM for **1k** in comparison of 3.40 μM for **1c**). In addition, **1k** was found to be 2.2 times more active than the reference drug CQ (IC_50 _=_ _0.20 μM). In contrast, the addition of a phenyl in position 2 of the pyrrolo[1,2-*a*]quinoxaline moiety (compound **1 l**) reduced considerably the antimalarial activity on the W2 strain (IC_50 _=_ _1.39 μM).

The bis-pyrrolo[1,2-*a*]quinoxalines **1m–n**, which are linked with polyaminoalkyl chains on position 3 of the benzyl moieties, increased the antimalarial activity up to 35 and 68 times when compared to their non-substituted counterparts linked in position 4 (compounds **1b–c**). Thus, compound **1n** bearing a diaminopropylpiperazine linker exhibited the most potent antiplasmodial activity (IC_50 _=_ _0.05 μM) against the CQ-resistant strain (W2). Nevertheless, in these subseries in which the polyaminoalkyl side chain was anchored in position 3 of the benzyl ring, the introduction of a methoxy substituent on the pyrrolo[1,2-*a*]quinoxaline heterocycles (compounds **1o–p**) seemed to be less detrimental against drug-resistant strain W2 in comparison with their analogs **1d–i**. The bis-pyrrolo[1,2-*a*]quinoxaline **1r**, structural isomer of derivative **1c**, was also found to have a significant activity against the W2 strain (IC_50 _=_ _0.75 μM). In addition, the tris{*N*-[(pyrrolo[1,2-*a*]quinoxalin-4-yl)benzyl]-3-aminopropyl}amines **1s–t** showed similar antimalarial activities when compared with their bis-homologs **1d–i** (IC_50 _=_ _0.19–0.29 μM for **1s–t***versus* 0.13–0.44 μM for **1d–i**). Moreover, the replacement of the polyaminoalkyl linker by a 1,11-diamino-3,6,9-trioxaundecane chain (compound **1u**) showed the same level of antimalarial activity against the W2 strain with IC_50 _=_ _0.25 μM.

Against the CQ-sensitive strain (3D7)and in the subseries of the pyrrolo[1,2-*a*]quinoxalines unsubstituted on their heterocyclic moieties (compounds **1a–c**), compound **1c** joined by a bis-(3-aminopropyl)piperazine linker was found more active than its structural analogs with bis-(3-aminopropyl)amine (compound **1a**) or bis-(3-aminopropyl)methylamine linkages (compound **1b**): i.e. IC_50 _=_ _0.80 μM for **1c***versus* 3.42 and 1.20 μM for **1a** and **1b**, respectively. Surprisingly, most of the pyrrolo[1,2-*a*]quinoxalines **1d–g** and **1i** bearing a methoxy substituent on the heterocyclic skeleton exhibited moderate antimalarial activity against 3D7 strain with IC_50_ ranging from 1.28 to 5.85 μM, in the exception of compound **1 h** which showed an interesting IC_50_ of 0.25 μM against this *Plasmodium* strain. As against the W2 strain, replacement of the methoxy function by an ethoxy one in position 3 of the pyrrolo[1,2-*a*]quinoxaline moieties (compounds **1j–k**) increased the anti *P. falciparum* activity against the 3D7 strain (IC_50 _=_ _0.26 and 0.19 μM for **1j** and **1k**, respectively). The introduction of a phenyl ring in position 2 of the pyrrolo[1,2-*a*]quinoxaline moiety (compound **1 l**) reduced considerably the antimalarial activity on the 3D7 strain (IC_50 _=_ _8.96 μM). Similar observations could be made with the structural analogs **1q–r** which showed moderate activity against 3D7 strain (IC_50 _=_ _1.00–7.22 μM). In the subseries in which the polyaminoalkyl side chain was anchored in position 3 of the benzyl ring, the piperazine substituted bis-pyrrolo[1,2-*a*]quinoxaline **1p** was found to be two times more active than the reference drugs chloroquine and mefloquine (IC_50 _=_ _0.040 μM for **1p***versus* IC_50 _=_ _0.080 μM for the references). The tris-pyrrolo[1,2-*a*]quinoxalines **1s–t** showed similar antimalarial activities when compared with their methoxy substituted bis-homologs **1o–p** (IC_50 _=_ _0.20–0.24 μM for **1s–t** versus 0.04–0.18 μM for **1o–p**). Compound **1u** bearing diaminotrioxaundecane linkage exhibited similar activity against the 3D7 strain than those observed for chloroquine and mefloquine (IC_50_–0.08 μM).

#### In vitro antileishmanial activity against promastigote forms

In order to increase the biological profile of molecules **1a–u**, complementary analyses were performed. Notably, *P. falciparum* belonging to coccidian protozoan parasites, their *in vitro* biological activity on flagellate protozoan parasites like *Leishmania donovani* was evaluated (Table 2). In comparison with amphotericin B and pentamidine used as reference drugs (IC_50 _=_ _0.1 μM and 5.5 μM, respectively), most of the tested compounds **1** were found active against the promastigote forms of *L. donovani*. It must be noticed that a majority of these bis-pyrrolo[1,2-*a*]quinoxaline derivatives **1** (compounds **1a**, **1c**, **1d–j** and **1m–q**)with IC_50_ ranging from 1.0 to 4.26 μM were found slightly more potent than pentamidine, one of the reference compounds (IC_50 _=_ _5.5 μM). In particular, bis{*N*-[4-(indolo[1,2-*a*]quinoxalin-6-yl)benzyl]-3-aminopropyl}piperazine **1q** was found to be 9.65 times more active than pentamidine (IC_50 _=_ _0.57 μM *versus* IC_50 _=_ _5.5 μM). In our previously described antileishmanial series^21^^,^^22^, we have noticed that introduction of a methoxy group in positions 7 and 8 of the pyrrolo[1,2-*a*]quinoxaline moiety generally increased the antileishmanial activity. In this new series, we also decided to introduce a methoxy or an ethoxy to evaluate the influence of such groups for their *in vitro* antileishmanial activity upon the *L. donovani* strain. Meanwhile, the presence of these alkoxy groups in the 7, 8, 9 or 3 position seems not to be detrimental for the activity against *L. donovani* as illustrated by results obtained for compounds **1d–k** and **1o–p** (IC_50 _=_ _1.07–4.26 μM) in comparison with unsubstituted compounds **1a**, **1c** and **1m–n** (IC_50 _=_ _1.0–3.71 μM). The same observation could be done for the nature of the polyalkylamine linker between the two benzylpyrrolo[1,2-*a*]quinoxaline moieties that seemed also not to be crucial, with the exception of piperazine substituted **1 g**, which showed a better antileishmanial activity *in vitro* compared to its structural methylamine analog **1f** (IC_50 _=_ _4.25 μM *versus* 1.07 μM for **1 g**) upon the *L. donovani* strain.

Moreover, in the subseries in which the polyaminoalkyl side chain was anchored in position 3 of the benzyl ring, the various bis-pyrrolo[1,2-*a*]quinoxalines **1m–p** were found less beneficial for the *in vitro* antiparasitic activity with an IC_50_ of 2.80–3.71 μM in comparison with their analogs **1a–c** and **1f–g**. In addition, the tri-substitution of the tris(3-aminopropyl)amine linker by the pyrrolo[1,2-*a*]quinoxaline core (compounds **1s–t**) led to a decrease in the antileishmanial activity (IC_50 _>_ _10 μM) in comparison with their bis-pyrrolo[1,2-*a*]quinoxaline analogs **1d–e** and **1o–p**.

#### Cytotoxicity and selectivity index

In order to assess their selectivity of action, the cytotoxicity of the antimalarial compounds **1a-u** was also evaluated *in vitro* upon one human cell line. The cytotoxic concentrations 50% (CC_50_) on the metabolizing human hepatocyte HepG2 cell line allowed access to the corresponding selectivity indexes (SI) defined as ratio of cytotoxic and antimalarial activities (SI = CC_50_/W2 or 3D7 IC_50_). The SI could validate their real potential as selective antiparasitic agents. The results concerning the cytotoxicity and SI data are presented in Table 2. As expected, most of the active bis- and trispyrrolo[1,2-*a*]quinoxalines **1** showed significant level of cytotoxicity against the HepG2 cell line (IC_50 _=_ _1.25–3.38 μM). Considering the W2 *P. falciparum* strain, the selectivity indexes varied between 0.37 and 40.6, and SI ranged from 0.53 to 39.25 by taking into account the 3D7 strain. This SI led to the identification of compound **1n** with SI of 40.6 on W2 strain, and SI of 39.25 for derivative **1p** on 3D7 strain. We could notice that these last bioactive compounds **1n** and **1p** were bis-pyrrolo[1,2-*a*]quinoxalines linked by a 1,4-bis(3-aminopropyl)piperazine moiety in position 3 of their benzyl ring. On the other hand, the bis-pyrrolo[1,2-*a*]quinoxaline **1u** is also interesting with SI =20.26, on the 3D7 malaria strain. However, according to the SI values, these compounds appeared to be toxic; thus, new modulations by replacing the benzyl substituted heterocyclic moiety to synthesize new candidates could be developed for forthcoming pharmacological investigations.

#### FRET-melting experiments

**Figure 2.**
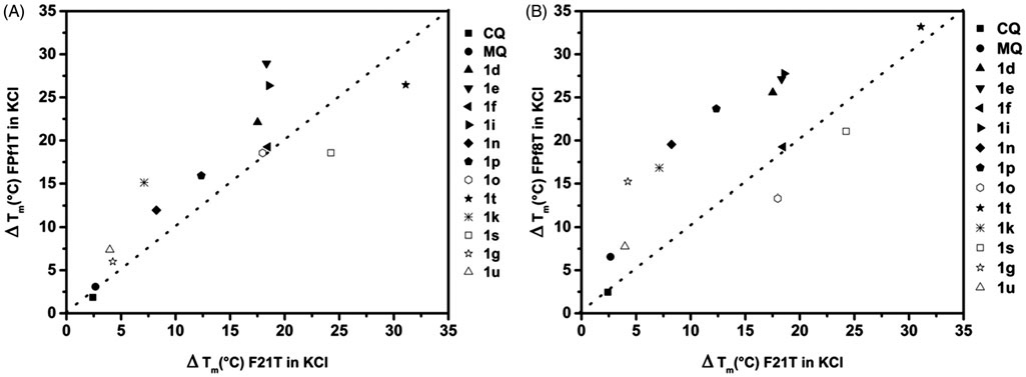
(A and B) Thermal stabilization (ΔT_m_) induced by different compounds **1** (at 5 μM) on the *Plasmodium* telomeric quadruplexes FPf1T and FPf8T *versus* ΔT_m_ induced on the human telomeric quadruplex F21T.

As these new compounds presented the same requirements as those required for G-4 stabilizing ligands, we have also investigated their ability of targeting *P. falciparum* telomeres as a potential strategy to interfere with human protozoan parasite infections. In fact, the telomeres of the parasite could constitute an attractive target. They are composed of repetitions of a degenerate motif (^5′^GGGTTYA^3′^, where Y could be T or C), which is different from the human one (^5′^GGGTTA^3′^). This antimalarial approach based on targeting *P. falciparum* telomeres has been previously described by our team^36^. Thus, in this report, we investigated the stabilization of *Plasmodium* telomeric G-quadruplexes by these new compounds through a FRET melting assay. For this evaluation, the two *Plasmodium* telomeric sequences (FPf1T and FPf8T) and the human one (F21T), in potassium, were used (two different *Plasmodium* telomeric repeats were used to fit the degenerate consensus). Thus, we used this FRET melting assay to study the interactions of some of our most bioactive compounds **1** and also the reference drugs (chloroquine – CQ and mefloquine – MQ) with these three telomeric sequences. For better visualization, a plot of ΔT_m_ values induced on FPf1T or FPf8T *versus* the ΔT_m_ induced on F21T is presented in Figure 2, allowing us to classify the more active compounds **1** in various subsets. Almost all the tested compounds displayed roughly the same stabilization profiles for the two *Plasmodium* telomeric sequences FPf1T and FPf8T and slightly better than that of the human one F21T, except **1s** and **1t** for FPf1T and **1o** and **1s** for FPf8T.The reference compounds CQ and MQ, never evaluated on these parasitic sequences before our work, showed very slight stabilization on these three telomeric sequences. These preliminary results mean that most of the tested bis-pyrrolo[1,2-*a*]quinoxalines **1** have a moderate preference for *Plasmodium* over human telomeric quadruplexes, excepted bispyrroloquinoxaline **1o**. Surprisingly, the tris-pyrrolo[1,2-*a*]quinoxalines **1s–t** were found more active on the human telomeric sequence F21T in comparison with their bis-analogues**1d–e** and **1o–p**. In contrast, the bis{*N*-[4-(methoxypyrrolo[1,2-*a*]quinoxalin-4-yl)benzyl]-3-aminopropyl}piperazines **1e** and **1i** were found to be more active on the *Plasmodium* sequences with the higher ΔT_m_. These last data could benow considered as a promising starting point for the further development and optimization of new and potent antimalarial compounds as well as promising stabilizers for the telomeric *Plasmodium* DNA sequences. As very little is known about telomere regulation in *P. falciparum*, further studies should be now investigated both on parasitic and human sequences which presented different response or time-response to telomere perturbation.

## Conclusion

In the present report, we have described the synthesis and the antimalarial activity of new bis- and trispyrrolo[1,2-*a*]quinoxaline derivatives **1** in which aromatic nuclei are joined by various aliphatic polyamines linker. These new compounds were then tested for their *in vitro* antiparasitic activity on (i) two *P. falciparum* strains (the CQ-resistant W2 and CQ-sensitive 3D7); and (ii) the promastigote forms of *L. donovani* strain. Among these new synthesized molecules, few of them were identified as potential *in vitro* antiplasmodial hits, displaying IC_50_ ranging from 0.04 to 0.09 μM on the W2 and 3D7 strains of *P. falciparum*. Thus, the most promising antimalarial results were obtained for the two bis-pyrrolo[1,2-*a*]quinoxalines **1n** and **1p** linked by a 3-(aminopropyl)piperazine chain in position 3 of their benzyl moieties. These compounds **1n** and **1p** were identified as the most potent antimalarial candidates with selectivity index (SI) of 40.6 on W2 strain, and 39.25 on 3D7 strain, respectively. In addition, biological results showed activity against the promastigote forms of *L. donovani* with IC_50_ ranging from 0.57 to 4.26 μM. In parallel, the *in vitro* cytotoxicity of these new molecules was assessed on the human HepG2 cell line. Structure-activity relationships of these new synthetic compounds are here also discussed, as well as their relative ability of targeting *P. falciparum* telomeres as potential mechanism of action. Thus, as the telomeres of the parasite could constitute an interesting target, we have also established the possibility of targeting *Plasmodium* telomeres by stabilizing the *Plasmodium* telomeric G-quadruplexes through a FRET melting assay. These results led us to conclude that the two bis{*N*-[4-(methoxypyrrolo[1,2-*a*]quinoxalin-4-yl)benzyl]-3-aminopropyl}piperazines **1e** and **1i** seemed to be able to discriminate between *Plasmodium* and human telomeric quadruplexes. By taking into account the biological and biophysical results and also the structure–activity relationships of these new series and those previously described, our overall results could be now considered as a promising starting point for the further development and optimization of new and potent antiprotozoan compounds.
